# The Many Roles of Ubiquitin in NF-κB Signaling

**DOI:** 10.3390/biomedicines6020043

**Published:** 2018-04-10

**Authors:** Gilles Courtois, Marie-Odile Fauvarque

**Affiliations:** INSERM U1038/BGE/BIG, CEA Grenoble, 38054 Grenoble, France; marie-odile.fauvarque@cea.fr

**Keywords:** nuclear factor κB, signal transduction, ubiquitin, ubiquitination/deubiquitination

## Abstract

The nuclear factor κB (NF-κB) signaling pathway ubiquitously controls cell growth and survival in basic conditions as well as rapid resetting of cellular functions following environment changes or pathogenic insults. Moreover, its deregulation is frequently observed during cell transformation, chronic inflammation or autoimmunity. Understanding how it is properly regulated therefore is a prerequisite to managing these adverse situations. Over the last years evidence has accumulated showing that ubiquitination is a key process in NF-κB activation and its resolution. Here, we examine the various functions of ubiquitin in NF-κB signaling and more specifically, how it controls signal transduction at the molecular level and impacts in vivo on NF-κB regulated cellular processes.

## 1. Introduction

Regulation of gene expression in eukaryotic cells represents an essential process for the timely control of the production of proteins, while the fine-tuning of their final activities and/or fate often relies upon post-translational modifications (PTM). For decades, phosphorylation and dephosphorylation has been considered as the dominant switch controlling the fate and activity of proteins. In the eighties, ubiquitination was identified as an additional type of PTM, although primarily in the restricted context of protein degradation. Later, the ubiquitination/deubiquitination process was shown to expand to the regulation of almost any protein, with its own intricate modulation allowing the emergence of new layers of regulation.

Among the many cellular processes that are regulated by ubiquitination is signal transduction which needs to be tightly controlled for operating properly at the right place and time. One of the first identified and most extensively dissected signaling pathways regulated by ubiquitination is the NF-κB pathway that plays a critical role in inflammation, immunity and control of cell death and proliferation. Not surprisingly, this pathway represents a useful paradigm to illustrate how ubiquitination controls protein activity. Here, we will present the main participants in the NF-κB activation process, how ubiquitination generally operates and which specific steps of this process it regulates. Moreover, we will describe how perturbations in ubiquitination and ubiquitin recognition mechanisms in the NF-κB pathway impact on human health.

## 2. The NF-κB Signaling Pathway

**Figure 1:**
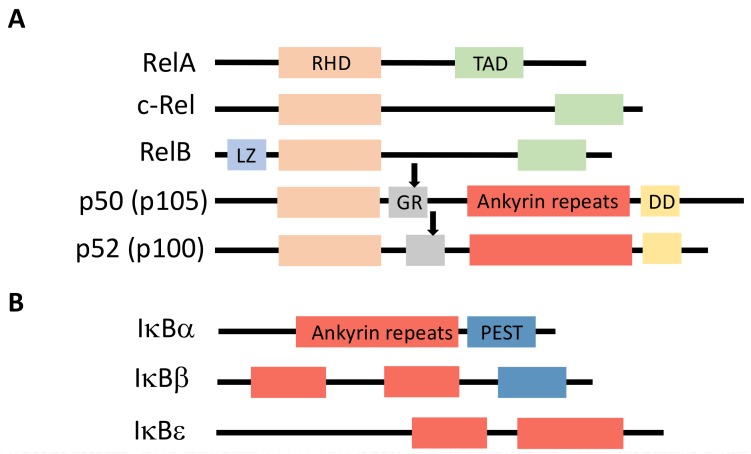
The NF-κB proteins and their inhibitors. (**A**) Members of the Rel/NF-κB family. The five NF-κB subunits are presented with their functional domains. RHD = Rel homology domain; TAD = transcription activation domain; LZ = leucine zipper; GR = glycine-rich domain; DD = death domain. The bold arrows indicate the C-terminus of p50 and p52 after processing of p105 and p100, respectively. (**B**) Members of the IκB family. The three IκB inhibitors are presented with their functional domains. PEST = proline/glutamic acid/serine/threonine-rich sequence.

NF-κB is a generic name for a collection of inducible transcription factors formed by the dimeric combination of members of the avian reticuloendotheliosis (Rel)/NF-κB family of proteins. The five members of this family (RelA, RelB, c-Rel, p50 and p52) share a conserved Rel homology domain at the N-terminus, which contains sequences involved in dimerization, nuclear localization and interaction with NF-κB inhibitors [1] (Figure 1A). In addition, RelA, RelB and c-Rel exhibit a transcriptional activator domain (TAD) at the C-terminus. Such a domain is absent in p50 and p52, which are both synthesized from precursors p105 and p100, respectively.

NF-κB regulates the transcription of hundreds of genes participating in immunity, inflammation, cell proliferation and cell death and its activity is itself controlled by a plethora of stimuli such as pro-inflammatory cytokines, pathogen-associated molecular patterns (PAMPs), and oxidative stress [2]. Fast activation of NF-κB is achieved through its ubiquitous presence in the cytoplasm of resting cells as a latent form associated with inhibitors of the inhibitory κB (IκB) or IκB-like families, and its quick release from them in response to stimuli to become transcriptionally competent.

Members of the IκB family of NF-κB inhibitors are IκBα, IκBβ and IκBε (Figure 1B). They all share a similar structure with a conserved sequence at the N-terminus containing a DSGXXS motif and a series of Ankyrin repeats at the C-terminus. These repeats are responsible for the interaction with the Rel domain of NF-κB proteins and the masking of their nuclear localization sequence (NLS). Precursor proteins p105 and p100 also contain Ankyrin repeats at the C-terminus and can play the role, before processing to generate p50 and p52, of IκB-like proteins.

Activation of NF-κB can be achieved through two distinct modes designated as “canonical” and “non-canonical”.

### 2.1. The Canonical Pathway of NF-κB Activation

**Figure 2:**
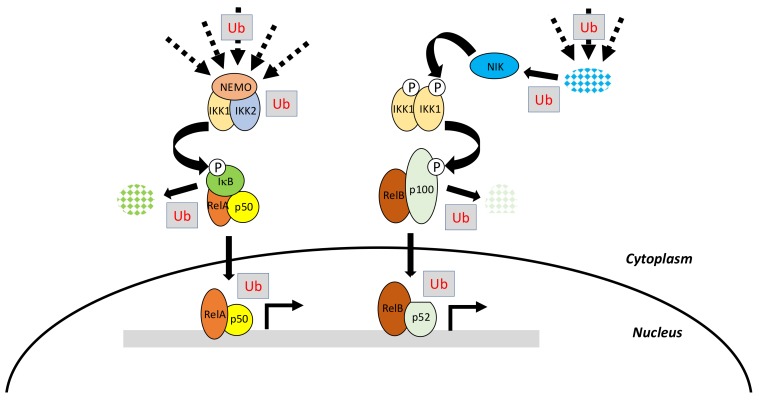
The canonical and non-canonical pathways of NF-κB activation. On the left is presented the canonical pathway which involves phosphorylation of IκBs by IKK to induce their degradation. On the right is presented the non-canonical pathway which is dependent on NIK stabilization and IKK1 activation. In each case specific NF-κB dimers are induced that regulates different classes of genes participating in various biological processes. Steps that are controlled by ubiquitination processes are indicated by “Ub”. See text for details.

**Figure 3:**
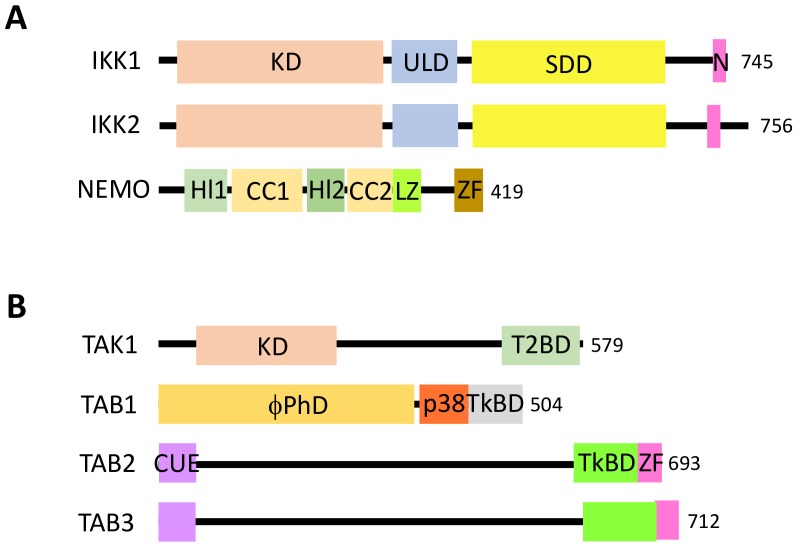
The subunits of IKK and TAK1 complexes. (**A**) IKK complex. The three subunits of this complex are presented with their functional domains. KD = kinase domain; ULD = ubiquitin-like domain; SDD = scaffold/dimerization domain; N = NEMO binding domain; Hl1/Hl2 = Helix 1 and 2; CC1/CC2 = coiled coil 1 and 2; LZ = leucine zipper; ZF = zinc finger; (**B**) TAK1 complex. The three subunits of this complex are presented with their functional domains. KD = kinase domain; T2BD = TAB2/TAB3-binding domain; φPhD = pseudo-phosphatase domain; p38 = p38-interacting domain; TkBD = TAK1-binding domain; CUE = coupling of ubiquitin conjugation to ER degradation domain; ZF = novel zinc finger (Npl4 class).

The canonical pathway, which is induced by a large variety of external or internal cell stimuli (see below), involves a cytoplasmic kinase complex called IκB kinase (IKK) (Figure 2). This complex is composed of three main subunits: two catalytic ones with related structures, IKK1 (also called IKKα) and IKK2 (also called IKKβ), and a regulatory subunit, NF-κB essential modulator (NEMO) (also called IKKγ) [3] (Figure 3A). Upon cell activation, IKK phosphorylates the two Serine residues located in the DSGXXS motif of IκB inhibitors and this induces their degradation by the proteasome (see details below). Free NF-κB (usually dimers such as RelA/p50, RelA/RelA or c-Rel/p50) translocate in the nucleus and positively or negatively regulate the transcription of numerous target genes, encoding proteins mostly involved in immunity, inflammation, cell growth and cell survival.

In this pathway, IKK activation is often triggered by another kinase complex, the tumor growth factor β-activated kinase 1 (TAK1) complex, that contains, in addition to the kinase TAK1, three regulatory subunits: TAK1 binding protein 1 (TAB1), which regulates the catalytic activity of TAK1, and TAB2 and TAB3, which participate in the activation of TAK1 by binding to ubiquitin (see below) [4] (Figure 3B).

### 2.2. The Non-Canonical Pathway of NF-κB Activation

The non-canonical pathway is an alternative pathway which is activated by a limited set of stimuli (see below) and requires a cytoplasmic kinase called NF-κB inducing kinase (NIK) [5]. Following cell stimulation, NIK, which is normally constitutively degraded, starts to accumulate and further phosphorylates an IKK1 dimer, which in this setting, works independently from NEMO and IKK2 (Figure 2). Activated IKK1 then acts on p100 precursor, commonly bound to RelB, to induce its processing to p52. Active p52/RelB dimer translocate in the nucleus and regulate transcription of a set of specific genes distinct from those regulated by the canonical pathway.

Initially, these two modules of NF-κB activation may appear quite simple. Nevertheless, they require, in addition to phosphorylation, the linkage of various kinds of ubiquitin moieties on a number of the main actors of these pathways at various steps (Figure 2). This feature substantially complicates the picture and provides further layers of specificity and regulation that play a major role in proper cell physiology. Before discussing in depth how ubiquitination impacts on NF-κB signaling we will briefly present the basics of ubiquitin molecular machinery.

## 3. Ubiquitination: Players and Mechanisms

### 3.1. The Ubiquitination Process

**Figure 4:**
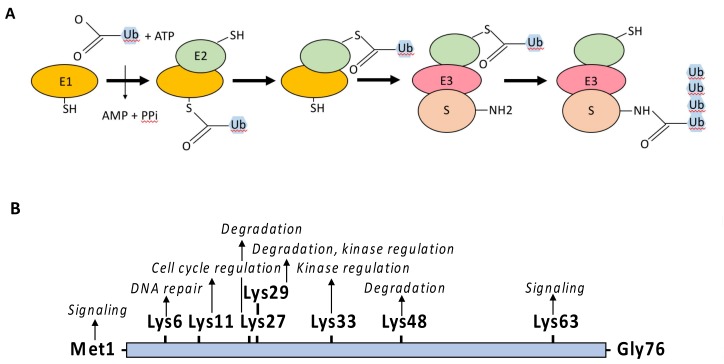
The ubiquitination process. (**A**) The enzymatic machinery. The three components (E1/E2/E3) involved in substrate (S) polyubiquitination and major steps of the ubiquitination process are shown; (**B**) key amino acids of ubiquitin. Indicated are Met1 and the seven internal Lys that can be used to form polyubiquitin chains through peptide (Met) or isopeptide (Lys) bonds involving Gly76. Main biological functions of these chains are indicated. See text for details.

Ubiquitin is an evolutionary conserved polypeptide of 76 amino acids that can be covalently attached through its terminal Gly residue to either the ε-amino group of a Lys residue (K) or to the amino group of the first Met (M1) of a protein target [6,7,8]. The linkage of ubiquitin depends on the successive action of E1 activating enzymes, E2 conjugating enzymes and E3 ligases (Figure 4A). The E1 catalyzes the ATP-dependent formation of a thioester bound between the C-terminus of ubiquitin and the active cysteine residue of E1. The ubiquitin is then trans-thiolated to the active cysteine of an E2 [9] and eventually transferred from the E2 to the target protein through the additional contribution of an E3 ligase. In this process, the E2–E3 complex is the main factor determining the specificity of the substrate. E3 ligases are quite diverse [10,11] but most of them possess a RING-finger domain and act as a bridging factor between the E2 and the substrate allowing the direct transfer of ubiquitin from the E2 to the target protein. Alternatively, E3 ligases possessing a homologous to the E6-AP carboxyl terminus (HECT) domain combine E2 and E3 activities and form a thioester intermediate with the active-site cysteine of the E3 prior to ubiquitin transfer to the substrate protein.

Ubiquitin itself contains seven Lys residues allowing for the formation of different ubiquitin polymerized chains by extension of the E2/E3 reaction or through the action of E4 enzymes [12] (Figure 4B). While the linkage of lysine-48-linked ubiquitin chains (Ub^K48^) drives proteins for degradation by the proteasome, other ubiquitin moieties, such as linear (M1) linked chains, Ub^K63^ linked chains or ubiquitin monomers, regulate protein activity, protein subcellular localization or protein-protein interaction in a plethora of cellular processes, including endocytosis, signal transduction or DNA repair [13].

At the structural level, ubiquitin displays a β-grasp fold and possesses a hydrophobic surface patch which mediates the interaction with ubiquitin-binding domains (UBDs) containing proteins (see below). Ubiquitin is itself subjected to other kinds of PTMs including the linkage of ubiquitin-like proteins (ULPs) (see below), phosphorylation and acetylation, adding further structural complexity to the so-called “ubiquitin code” [14].

Ubiquitin proteases, also known as deubiquitinases (DUBs) catalyse the reverse reaction by hydrolysing ubiquitin from protein or polyubiquitin chains [15]. When acting at the level of the proteasome, they favour protein entry into the proteasome machinery and ubiquitin recycling. Alternatively, DUBs can act at earlier steps and save protein from degradation or interfere with any cell process. Mammalian genomes contain almost one hundred DUBs belonging to two main classes of proteases: the metalloproteases (JAB1/MPN/Mv34 metalloenzymes (JAMMs)) and the cysteine proteases (ubiquitin specific protease (USP), ubiquitin C-terminal hydrolases (UCHs), MIU-containing novel DUB family (Mindy), Machado-Joseph disease proteases (MJDs), ovarian tumor proteases (OTUs)], among which the USP subfamily represents the largest class [16]. DUBs contain a catalytic domain that has sequence similarity within subfamilies and unrelated flanking sequences that typically mediate protein-protein interaction and/or regulate the catalytic activity of the enzyme as shown for a number of DUBs containing ubiquitin-like domains (ULDs) that share a structure similar to ubiquitin. These flanking sequences, along with the catalytic core, can also contribute to the specific binding and cleavage of different polyubiquitin chains (see below). Actually, DUBs activity and substrate specificity are governed by many factors that are not fully elucidated and certainly depend on subcellular localization and target recognition through the integration of DUBs into large protein complexes [17]. Notably, DUBs and E3 ligases targeting a common substrate sometimes act within the same protein complex, possibly finely regulating protein activity through a ubiquitination/deubiquitination cycle [18].

A number of ubiquitin-like proteins (ULPs) have been reported [19], sharing a similar structure and conjugation mechanisms with ubiquitin, including the closely-related small ubiquitin-like modifier (SUMO), which plays an essential role in DNA repair, cell cycle, and signal transduction [20], and the neural precursor cell expressed, developmentally down-regulated 8 (NEDD8) [21], which modulates the function of the cell cycle and embryogenesis proteins.

Importantly, the number of E3 Ligases or DUBs mutations found to be associated with human pathologies such as inflammatory diseases, rare diseases, cancers and neurodegenerative disorders is rapidly increasing [22,23,24]. There is now clear evidence that many E3s and DUBs play critical roles in NF-κB signaling, as will be discussed in the next sections, and therefore represent attractive pharmacological targets in the field of cancers and inflammation or rare diseases.

### 3.2. E3 Ligase Families in NF-κB Signaling

Specific classes of E3 ligases participating in the NF-κB signaling pathways described in the following sections deserve a short introduction.

#### 3.2.1. TRAFs

E3 ligases of the small TNF receptor associated factor (TRAF) family (seven members) [25] present a conserved organization with a really interesting new gene (RING) finger domain at the N-terminus, followed by a variable number of zinc fingers (ZFs) and in the second half a so-called TRAF domain, which is divided into two parts: the TRAF-N domain, which is a coiled-coil and the TRAF-C domain, with both of them participating in oligomerization and substrate recognition. Only two TRAFs lack one of these conserved domains: TRAF1, which does not contain any ring domain and behaves only as an inhibitor or adaptor and TRAF7, which does not contain a TRAF-C domain and therefore cannot necessarily be considered as a bona fide TRAF. The specific interaction of TRAFs with their targets often involves specific motifs recognized by the TRAF-C domains. PXQXT/S represents the consensus binding sequence for TRAF2, TRAF3 or TRAF5 whereas PXEXX acidic/aromatic is recognized by TRAF6.

#### 3.2.2. TRIMs

Tripartite motif proteins (TRIMs) belong to a large family of E3 ligases of more than 70 members which display a conserved ring, B-box, coiled-coil (RBCC) domain at the N-terminus [26,27]. This domain includes a RING domain with E3 ligase activity, one or two B-box domains, and a coiled-coil, with these two latter domains participating in dimerization and higher-order oligomerization. The variable C-terminal regions of TRIMs, classified into 11 distinct classes, regulate target recognition and subcellular localization.

#### 3.2.3. LUBAC

Linear ubiquitin chain assembly complex (LUBAC) is the only E3 ligase complex described so far that is able to synthesize M1-linked ubiquitin chains in mammalian cells [28,29]. It is composed of three subunits, haem-oxydized IRP2 ubiquitin Ligase 1L (HOIL-1L), HOIL-interacting protein (HOIP) and SHANK-associated RH domain interacting protein (SHARPIN), each of which exhibit specific functions. HOIP is the subunit that contains all the catalytic machinery to synthesize M1-linked chains of ubiquitin. This involves a ring between ring fingers (RBR) domain and the C-terminal linear ubiquitin chain determining domain (LDD), allowing HOIP to work as a RING/HECT hybrid E3.

HOIL-IL and SHARPIN are non-catalytic subunits that associate to the ubiquitin-associated (UBA) domain of HOIP through their ubiquitin-like (UBL) domains. The SHARPIN UBL domain may also recognize the Npl4 Zinc Finger 2 (NZF2) domain of HOIP. Other important sequences of LUBAC subunits are (1) the PNGase/UBA or UBX-containing proteins (PUB) domain of HOIP which interacts with DUBs acting in NF-κB signaling, OTU deubiquitinase with linear linkage specificity (OTULIN) and cylindromatosis (CYLD), directly in the former case and through SPATA2 in the latter, (2) the NZFs of HOIL-IL and SHARPIN, which recognize ubiquitin (see below), and (3) the Pleckstrin homology (PH) domain of SHARPIN, that acts as a dimerization module. Like HOIP, HOIL-IL contains an RBR domain at the C-terminus, but this domain is dispensable for catalysis in the context of LUBAC.

All these E3 ligases work with specific E2 conjugating enzymes to synthesize different kinds of ubiquitin chains during the NF-κB activation process. We will not describe them exhaustively in the following sections, mostly because they are much less characterized than the E3 ligases. Nevertheless, two of them should be mentioned. In many situations, if not all, the specific E2 conjugating K63-linked chains is ubiquitin-conjugating 13 (Ubc13) which forms a dimer with co-factor ubiquitin-conjugating enzyme variant 1A (Uev1A). Ubc13/Uev1A was originally identified as the E2 associated with TRAF6 in cell extracts used to study the activation of NF-κB [30] in vitro. Its broad involvement in NF-κB signaling has been confirmed in vivo [31]. In addition, the main E2 conjugating M1-linked chains to specific components of the NF-κB pathways (see below) with LUBAC is the ubiquitin-conjugating enzyme E2 L3 (UBE2L3) [32,33].

### 3.3. Ubiquitin Binding Domains in NF-κB Signaling

Interpretation of the “ubiquitin code” is achieved through the recognition of different kinds of ubiquitin moieties by specific UBD-containing proteins [34]. UBDs are quite diverse, belonging to more than twenty families, and their main characteristics can be summarized as follows: (1) They vary widely in size, amino acid sequences and three-dimensional structure; (2) The majority of them recognize the same hydrophobic patch on the β-sheet surface of ubiquitin, that includes Ile44, Leu8 and Val70; (3) Their affinity for ubiquitin is low (in the higher µM to lower mM range) but can be increased following polyubiquitination or through their repeated occurrence within a protein; (4) Using the topology of the ubiquitin chains, they discriminate between modified substrates to allow specific interactions or enzymatic processes. For instance, K11- and K48-linked chains adopt a rather closed conformation, whereas K63- or M1-linked chains are more elongated.

In the NF-κB signaling pathway, several key players such as TAB2/3, NEMO and LUBAC are UBD-containing proteins whose ability to recognize ubiquitin chains is at the heart of their functions.

Within the TAK1 complex, TAB2 and TAB3 are the UBD-containing subunits. They present a similar secondary structure to an N-terminal coupling of ubiquitin conjugation to endoplasmic reticulum-associated degradation (CUE) UBD, a coiled-coil, a TAK1-binding domain and, at the C-terminus, a NZF (Figure 3B). The NZF is responsible for the interaction with ubiquitin. Recognition of K63-linked ubiquitin chains requires binding of adjacent ubiquitin moieties by two binding sites, both of them involving the Ile44-containing hydrophobic patch [35,36]. The distal ubiquitin occupies the canonical NZF at residues Thr674 and Phe675 of TAB2, whereas the proximal one contacts residues Leu681, His678 and Glu685. As a consequence, the TAB2 NZF is surrounded by three ubiquitin molecules, two of them interacting with one NZF. This two-sided mode of interaction may be shared by other NZF-containing proteins but, very importantly, excludes recognition of M1-linked chains of ubiquitin in the case of TAB2/3.

Within the IKK complex, NEMO is the specialized subunit allowing protein scaffolding through the recognition of specific partners modified by ubiquitin. The interaction of NEMO with polyubiquitin involves two separate domains. First, the NEMO ubiquitin binding (NUB) domain, encompassing the CC2 and leucine zipper (LZ) domains, recognizes both M1- and K63-linked chains through distinct modes. Two M1-linked ubiquitin dimers can be recognized by the dimeric NUB domain through the interaction of the proximal ubiquitin (residues extending from Gln2 to Glu16 plus Glu64 and Thr66) with NEMO Arg and Glu residues located from 309 to 320 and interaction of the distal ubiquitin through its hydrophobic patch and NEMO residues centered around Asp304 [37]. This extended interaction interface, which also includes the linker region, explains the much higher affinity of the NUB domain for M1-linked chains than for K63-linked chains. Indeed, in the case of K63-linked chains, only the distal ubiquitin can be recognized because of a slight shift of the proximal ubiquitin caused by the K63 linkage [38]. Second, the ZF located at the very end of NEMO also displays affinity for ubiquitin, favoring recognition of K63-linked chains. Again, a two-sided interaction occurs, with the hydrophobic patch of distal ubiquitin binding to ZF residues Val414/Met415 and residue Phe395-centered patch connecting to the proximal ubiquitin [39]. It has been proposed that combined recognition of polyubiquitin chains by the NUB domain and the ZF occurs in the full-length protein [40], resulting in an affinity for both M1-linked and K63-linked chains, with a stronger affinity for the former ones [41]. This ability to recognize both kinds of chains may prove important considering the synthesis of mixed chains that can occur during cell stimulation (see below).

All LUBAC subunits contain similar NZFs of the Npl4 subtype but they appear to fulfill different functions. The NZF of HOIP displays a weak affinity for K63-linked chains compared to the ZF of SHARPIN; so SHARPIN is the driving force for recruitment to K63-linked partners [42]. Molecular characteristics of these NZF/ubiquitin interactions are not known. In contrast, HOIL-IL specifically recognizes M1-linked ubiquitin chains through a unique mode [43]. Indeed, its NZF binds both the canonical Ile44-centered hydrophobic surface on the distal ubiquitin and a Phe4-centered hydrophobic patch on the proximal ubiquitin. These distinct specificities help explaining how LUBAC operates within the NF-κB signaling pathways, as will be further detailed in Section 5.

## 4. Regulated Ubiquitination of IκBs and NF-κB Precursors

### 4.1. Regulated Ubiquitination of IκBs

**Figure 5:**
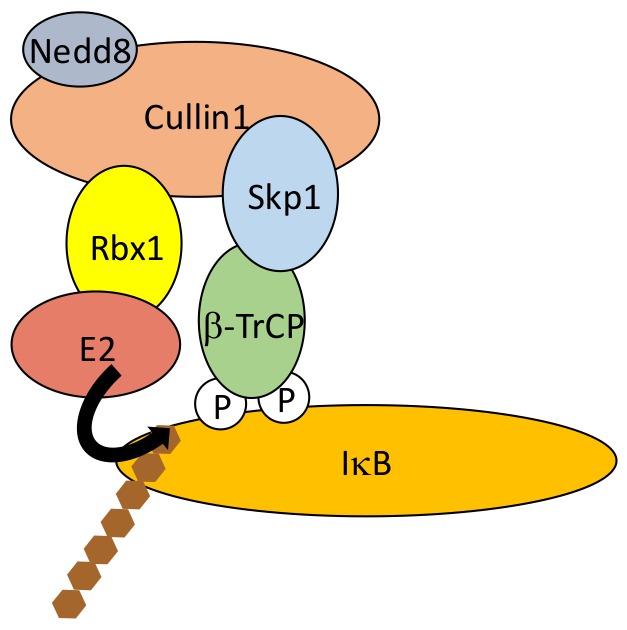
The degradation machinery of IκBs. The SCF E3 ligase complex that induces degradative ubiquitination of IκBs is depicted. K48-linked polyubiquitination is indicated with brown hexagons. See text for details.

As discussed above, the critical step in NF-κB activation is the phosphorylation-induced ubiquitination and degradation of IκBs, allowing NF-κB dimers to translocate into the nucleus. In the case of the three classic IκB proteins, IκBα, IκBβ and IκBε, a similar amino-acid sequence is targeted for phosphorylation by IKK. It includes two serine residues located within a DSGXXS motif [44] also found in other proteins whose activity is controlled by proteasome degradation such as β-catenin and mouse double minute 2 homolog (Mdm2). This “degron” is recognized by the E3 ligase Skp, Cullin, F-box (SCF) containing complex [45,46]. More specifically, phosphorylation of IκBs Serine residues (Ser32/36 for IκBα, Ser19/23 for IκBβ and Ser18/22 for IκBε) induces the recruitment of SCF through β-transducing repeat-containing protein (β-TrCP), an F-box/TrpAsp 40 aa (WD40)-repeat protein. Within the SCF complex β-TrCP interacts with S-phase kinase-associated protein 1 (Skp1), bringing other components such as Cullin, RING-box protein 1 (Rbx1) and the E2 conjugating enzymes UBCH5b, UBCH5c or cell division cycle 34 (CDC34)/UBC3 [47] in proximity to the end-terminus of IκB (Figure 5). This allows K48-linked chain addition to Lys21/22 of IκBα, Lys9 of IκBβ and Lys6 of IκBε for degradation by the 26S subunit of the proteasome. Differences exist between the degradation kinetics of IκBα, IκBβ and IκBε, and the generating waves of NF-κB dimers. This might be caused by differences in degrons environment influencing their phosphorylation by IKK [48].

The SCF activity is regulated by neddylation, a post-translational modification sharing similarities with ubiquitination (see above). The NEDD8-conjugated subunit is Cullin [49] and neddylation involves the E2 conjugating enzyme Ubc12, which helps recruit E3 ligases to SCF [50]. SCF activity can also be shut-off by E3 ligase TRIM9, at least in the brain [51]. E3 activity of TRIM9 is not required in this case. Instead, a phosphorylated degron within TRIM9 competes for β-TrCP binding to phosphorylated IκB. Finally, SCF components have been shown to be targeted by viral proteins to block NF-κB function in the anti-viral response. For instance, Non-Structural Protein 1 (NSP1), a rotavirus-derived protein induces the ubiquitination-dependent proteasomal degradation of β-TrCP [52].

Ubiquitination of IκBs is also attenuated by several DUBs, resulting in inhibition of the NF-κB activation process. First, USP11 has been shown to associate with IκBα and to catalyze its deubiquitination in the TNF-α signaling pathway [53]. Second, the constitutive photomorphogenesis 9 (COP9) signalosome, that regulates the assembly and activity of Cullin-E3 ligases, may inhibit sustained IκBα degradation by inducing its deubiquitination by associated USP15 [54]. Another ubiquitin moiety that may regulate IκBs degradation is monoubiquitination. Indeed, a pool of monoubiquitinated IκBα has been identified as insensitive to TNF-α-induced degradation through impaired phosphorylation [55]. Previously, it had been proposed that sumoylation of IκBα may also influence its stability or fate [56]. These processes remain poorly characterized, especially concerning the modified residues and the proteins involved.

### 4.2. Regulated Ubiquitination of p105

**Figure 6:**
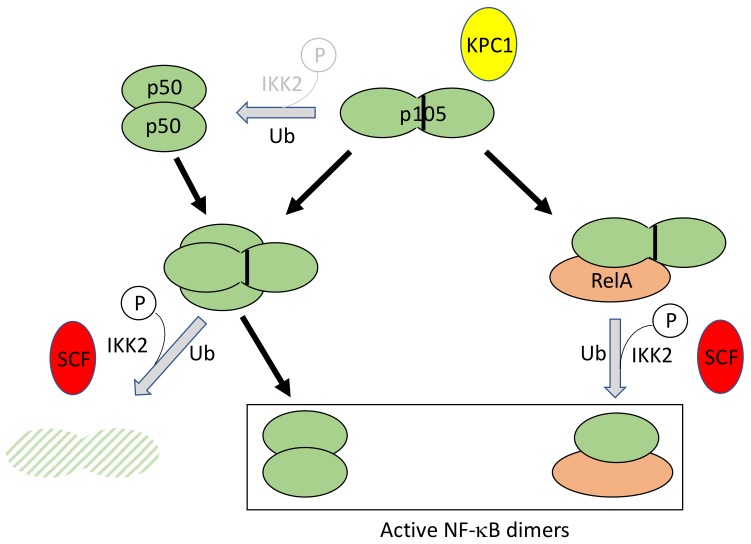
Regulated processing/degradation of p105. KPC1-dependent constitutive processing of p105 to generate p50, which can be slightly augmented upon IKK2 activation, is shown at the top. Complete proteolysis or limited processing to release active NF-κB dimers (p50/p50 or p50/RelA) upon cell activation is shown at the bottom. See text for details.

Processing of p105 to generate NF-κB subunit p50 occurs constitutively in resting cells, at low level, and can be amplified to a little extent upon stimulation. In addition, p105 associates with other NF-κB subunits, such as dimeric p50 or RelA, and can be either fully degraded or processed to p50 upon cell activation (Figure 6). Until now, how these various processes are coordinately regulated is still poorly understood, considering their dependency on the enzymatic machinery of the proteasome which is supposed to proteolyze its substrates to completion.

Originally, it was demonstrated that basal p105 processing to p50 required K48-linked ubiquitination and involved a long Gly-Ala repeat acting as a stop signal for degradation in the middle of the molecule [57]. Only recently the E3 ligase involved in p105 ubiquitination, Kipl ubiquitylation-promoting complex 1 (KPC1), has been identified upon chromatographic purification [58]. It has been shown to interact with p105 through its Ankyrin repeats. Interestingly, this interaction is increased upon phosphorylation of p105 at Ser927 by IKK2.

In addition, p105 can also be totally degraded upon cell stimulation, releasing its associated NF-κB dimers [59]. In this case, an IKK2-dependent phosphorylation first occurs at residues 927 and 932, located in sequences exhibiting similarities to the IκB degrons (see above), inducing recognition and ubiquitination by the SCF/β-TrCP complex [60,61].

It still remains to be understood how the processing versus degradation choice is made. It has been proposed that the Gly-Ala repeats preceding, during nibbling by the proteasome, a tightly folded domain at a precise distance would be sufficient to halt proteolysis of p105 [62], but how this can be bypassed remains unknown.

Finally, processing and degradation of p105 can be negatively regulated by the DUB A20. During processing, A20 can interact with p105 through KPC1 and inhibit its ubiquitination [63].

### 4.3. Regulated Ubiquitination of p100

In contrast to p105, the processing of p100 for generating p52 is exclusively inducible and dependent on IKK1 activation by NIK [64,65,66]. At the C-terminus of p100, a ^865^DSAYGS^870^ sequence is located, similar to the one found in IκB proteins. Upon its phosphorylation by IKK1, this sequence is recognized by SCF^TrCP^, inducing ubiquitination of p100 with K48-linked chains and processing to generate p52 [67,68]. As for p105, it is unclear how a limited proteolysis of p100 by the proteasome is achieved.

The steady-state level of p100 itself is controlled by another SCF complex: SCF^fbw7^ [69,70]. Precursor p100 contains two conserved sequences which exhibit similarities to the TPPLSP degron recognized by F-box/WD repeat-containing protein 7 (Fbw7), a member of the F-box family of proteins. Ser and Thr residues within these sequences are constitutively phosphorylated by glycogen synthase kinase-3 (GSK3). This induces p100 recognition by SCF^fbw7^ and K48-linked polyubiquitination, triggering p100 degradation by the proteasome. How this control of the p100 amount influences the non-canonical NF-κB pathway remains unclear. It may limit the amount of p52 produced in the cytoplasm. Alternatively, as proposed by Busino et al. [70], p100 elimination through SCF^fwb7^ may occur in the nucleus, decreasing the level of an inhibitory molecule and resulting in more efficient NF-κB activation.

In the following sections we will describe the major upstream signaling pathways activating NF-κB, focusing on ubiquitin-related events permitting and regulating signal transduction. The protein/protein interfaces that are involved will not be described in detail although they provide the primary level of specificity. Instead, excellent reviews dealing with molecular organization of the mentioned proteins will be referred to. Ubiquitin modifications affecting components of the TAK1 and IKK complexes represent shared features of most, if not all, of the signaling pathways described and will be presented in Section 6.

## 5. Regulated Ubiquitination during Intracellular Signal Transduction

### 5.1. The TNF-R1 Signaling Pathway

**Figure 7:**
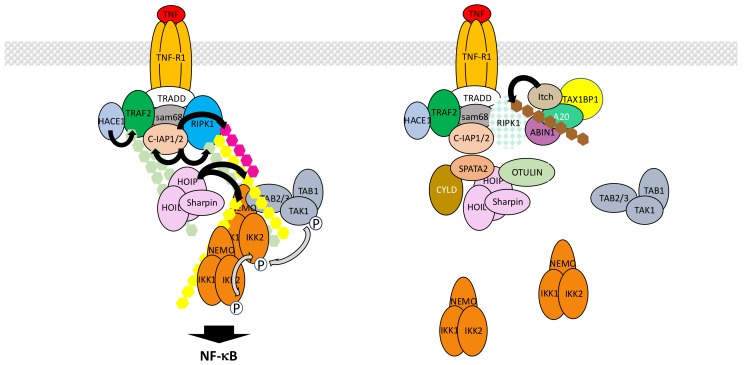
TNF-R1 signaling pathway. Components and mechanisms ensuring signal transduction in this pathway are depicted on the left, with black arrows indicating ubiquitination processes and grey arrows phosphorylation. M1-, K11- and K63-linked polyubiquitination is indicated with yellow, pink and green hexagons, respectively. Components and mechanisms participating in signal shut-off are presented on the right. An induced proteolysis of RIPK1, in addition to its deubiquitination, is indicated although its relevance in NF-κB signaling is uncertain. K48-linked polyubiquitination is indicated with brown hexagons. See text for details.

TNF-α is a pleiotropic inflammatory cytokine that binds to two distinct receptors, TNF-R1 and TNF-R2, with TNF-R1 exhibiting the broader cellular distribution. The TNF-R1 signaling pathway is by far the most extensively analyzed NF-κB activation pathway and provides the best integrated example to illustrate how various ubiquitination processes contribute to the formation of multiprotein complexes and triggers signal transduction. So far, at least a dozen of distinct proteins has been involved in the building and activity of the so-called TNF-R1 complex 1 that initiates NF-κB signaling (Figure 7). They are all heavily regulated by PTMs, many of them involving ubiquitin. These modifications, which require several E3 ligases operating at distinct levels, generate numerous active interfaces through UBDs. Equally important is the role of negative regulators, mostly of the deubiquitinase family, that ensure the fine-tuning of signal transduction, controlling both the level and the duration of the activation process.

Upon TNF-α exposure TNF-R1 trimerizes and recruits the adaptor tumor necrosis factor receptor type 1-associated death domain protein (TRADD) at its intracytoplasmic domain [71,72]. This allows TRADD to attract both kinase receptor-interacting serine/threonine-protein kinase 1 (RIPK1) through its death domain and E3 ligase TRAF2 through its N-terminus, with the possible participation of adaptor Src-associated in mitosis 68 kDa (Sam68) [73]. Subsequent polyubiquitination of RIPK1 is a key node event in signal transmission [74]. Unexpectedly, TRAF2 does not behave as the RIPK1 E3 ligase [75] in this setting. Instead, it plays a role as a scaffold protein recruiting two other E3 ligases, cellular inhibitor of apoptosis protein-1 (c-IAP1) and c-IAP2, that add K63- and K11-linked chains to RIPK1. This TRAF2/c-IAP1/2 interaction and the resulting polyubiquitination of RIPK1 triggers the recruitment of the E3 LUBAC complex that targets several components of the complex 1, including RIPK1 and c-IAPs themselves, through the addition of M1-linked ubiquitin chains [76]. At this stage, polyubiquitinated RIPK1 attracts kinase complexes TAK1 and IKK, through their UBD-containing subunits TAB2/TAB3 and NEMO, respectively. This eventually triggers IKK phosphorylation by TAK1 and NF-κB activation. Importantly, although a model describing K63-linked ubiquitin chains attracting TAK1 through TAB2/TAB3 on one side and M1-linked chains attracting IKK through NEMO on the other was originally proposed, the recent discovery of synthesized mixed polyubiquitinated chains [77] suggests instead an even more promiscuous mechanism with optimal proximity between IKK and its kinase TAK1 [78], further amplified by LUBAC-induced M1-linked ubiquitination of NEMO itself inducing IKK auto-aggregation.

This set of events represents a consensual basic model of IKK/NF-κB activation upon TNF-α exposure (see below for its limitations and several controversial issues). In addition, several other layers of regulation are likely to operate, involving a collection of components. At this stage, it is still difficult to unequivocally distinguish their real contribution to the NF-κB activation process from their role in regulating the so-called RIPK1 «switch» that control the cell survival versus death decision [79]. Indeed, upon TNF-α exposure a cell is subject to a distinct fate depending on the post-translational status of RIPK1 and its interaction with specific partners. As discussed above, ubiquitination of RIPK1 in complex 1 at the cell membrane contributes to NF-κB activation by allowing recruitment of IKK and its activating kinase TAK1. Nevertheless, RIPK1 is also a critical component of the cytoplasmic complex 2 that forms with pro-caspase 8 and Fas-associated protein with death domain (FADD) upon TNF-α exposure and triggers apoptotic cell death. In the case of NF-κB activation, the complex 2 is kept inactive through the neutralization of pro-caspase 8, while in contrast in the absence of NF-κB activation, caspase 8 is activated. An additional NF-κB-related brake on complex 2 activation is the phosphorylation of RIPK1 by IKK [80]. Another RIPK1-containing complex (complex 3) can also form, containing the related protein RIPK3, when caspase 8 or FADD activity is abolished. It induces death through necroptosis and involves mixed lineage kinase domain-like pseudokinase (MLKL), a substrate of RIPK3 inducing membrane pores and cell lysis [81].

Among the regulators that affect ubiquitin-related events in NF-κB activation but also control the RIPK1 switch are several DUBs [82]. The first to be identified was A20, also known as TNF alpha induced protein 3 (TNFAIP3), which is encoded by an NF-κB regulated gene and is a member of the OTU family of deubiquitinases. It has been claimed that it also exhibits E3 ligase activity [83] but it remains unclear whether this is an intrinsic activity within cells or an activity due to its interaction with E3 ligases such as Itch and RING finger protein 11 (RNF11) [84]. As a consequence, A20 can deubiquitinate the K63-linked chains of RIPK1 but may also regulate its stability through direct or indirect induction of K48-linked polyubiquitination. Its own ubiquitin protease activity appears regulated by IKK2-induced phosphorylation [85] providing another regulatory link between this enzyme and the NF-κB signaling pathway. Finally, A20 interacts with A20-binding inhibitor of NF-κB activation 1 (ABIN1), a protein with an affinity for ubiquitin, and this interaction plays a critical function in its recruitment to TNF-R1 complex 1 and RIPK1 deubiquitination [86]. To what extent A20 impacts on NF-κB signaling remains uncertain. Wertz et al. [85] have shown that due to its specific affinity for K63-linked chains and the absence of M1-linked chains recognition by A20, it modestly affects the NF-κB activation process and acts mostly on TAK1 dependent signaling which also connects to MAPK pathways.

The second DUB regulating ubiquitination process upon TNF-α stimulation is CYLD. CYLD is a divergent member of the USP family that specifically hydrolyzes K63-linked or M1-linked chains [87]. To some extent its expression can be modulated by NF-κB but it seems to be already present in resting cells and could limit the activation process of NF-κB by deubiquitinating RIPK1 [88]. Other CYLD substrates may include its two direct partners NEMO or TRAF2 [89]. Recently, Spermatogenesis-associated protein 2 (SPATA2) has been identified as a critical protein for recruiting CYLD to LUBAC within complex 1 through HOIP interaction [90]. In several instances, often linked to cell transformation and cancer, an up-regulation of NF-κB activity has been associated with the decreased expression or activity of CYLD [91].

OTULIN is another DUB whose function has been extensively characterized in the context of TNF-R1 signaling. It is a member of the OTU family of DUBs that exhibits a very specific and strong affinity for M1-linked chains [92,93]. Consequently, it regulates LUBAC-induced ubiquitination after interacting with HOIP through a PUB/PIM interface [94,95]. Upon over-expression OTULIN blocks NF-κB activation, whereas its down-regulation results in amplified NF-κB activation, most likely by controlling the level of NEMO M1-linked ubiquitination.

Other USPs may also participate in the NF-κB activation process in response to TNF-α but their exact mode of action and specific target(s) remain uncertain. USP2 has been shown to negatively regulate NF-κB activity in one case [96] and positively in another [97]. Again, the level of RIPK1 ubiquitination has been shown to depend on this enzyme. Two other USPs have been reported to control the activity of the same target. First, USP4 negatively regulates TNF-α-induced NF-κB activation by removing K63-linked chains on RIPK1 upon interaction [98]. Incidentally, this DUB has been reported to also target TRAF2 [99] and additional components of the NF-κB signaling pathway (see Section 5.2 and Section 6.1). Second, USP21 also displays affinity for RIPK1 and acts as a RIPK1 deubiquitinase negatively regulating NF-κB activation [100]. Whether these DUBs cooperate and work in the same way in different cell types or cooperate remain unknown.

The activity/stability of other complex 1 components can also be regulated by ubiquitination processes. First, TRAF2 has been shown to be modified by K63-linked polyubiquitin upon TNF-α exposure and this requires its previous phosphorylation at Thr117 by protein kinase Cδ (PKCδ) and PKCε [101]. The E3 ligase involved may be HECT domain and ankyrin repeat containing E3 ubiquitin protein ligase 1 (HACE1) [102]. K63-linked polyubiquitination of TRAF2 would help recruit TAK1 and IKK. TRAF2 expression level can also be controlled by K48-linked phosphorylation for proteasome recognition. This may be achieved by the carboxy terminus of Hsc70 interacting protein (CHIP), a U-box-dependent E3 ligase that interacts with TRAF2 [103].

The regulation of c-IAPs amount and activity in the TNF-R1 signaling pathway is also controlled by ubiquitin-related events. These proteins exhibit potent E3 ligase activity and are able to ubiquinate a collection of partners (TRAF2 may be one of them) or to auto-ubiquitinate, for proteasomal degradation. This latter property, degradation through self-ubiquitination, can be exploited for therapeutic purpose with Smac mimetics [104]. The deubiquitinase OTUB1 modulates this step by disassembling K48-linked chains from c-IAPs [105]. USP19 has also been shown to interact with c-IAPs and to prevent c-IAP2 degradative ubiquitination, leading to protein stabilization [106]. It would be interesting to study how USP19 impacts on NF-κB, given its influence on apoptosis.

So far, nothing has been reported regarding the control of LUBAC stability during the normal TNF-R1 signaling process. Nevertheless, it is worth mentioning that invasion-plasmid antigen-H proteins 1.4/2.5 (IpaH1.4/2.5), *Shigella* modulators of innate immune signaling, blunt the NF-κB pathway by interacting with HOIL-1L and conjugating K48-linked chains to HOIP for degradation [107].

Despite the wealth of data regarding the components and mechanisms involved in NF-κB activation by TNF-α, important issues concerning the exact role of the key participants still need to be clarified. For instance, RIPK1 has been presented above as critical in IKK activation (and it is also crucial in cell death induction, but this is not the focus of this review) but specific situations in which it is not required for NF-κB activation have been reported. Indeed, Wong et al. [108] have shown that several murine cell types are still able to degrade IκBα in response to TNF-α when *Ripk1* is invalidated. One may therefore wonder whether what initially appears as a very elaborated molecular system for recruiting kinases to activate NF-κB, can be fairly well compensated by extensive polyubiquitination events occurring on other components of complex 1 such as TRAF2, c-IAPs and LUBAC, with only “subtle” effects on the timely regulation of the process. In this regard, Xu et al. [109] have shown, using an elegant ubiquitin replacement strategy, that K63-linked poly-ubiquitination is dispensable for TNF-α-mediated NF-κB activation. Thus, other modifications involving M1-linked ubiquitin chains may be sufficient to induce NF-κB, at least in cell culture conditions. Something in accord with this flexibility has been reported by Blackwell et al. [110]. Of course, these particularities may not apply to the RIPK1 “switch” controlling cell death.

The other important question relates to the true function of TRAF2 within complex 1. As mentioned above, TRAF2 is considered to play a role as a scaffold allowing the recruitment of c-IAPs for RIPK1 polyubiquitination. This is based on the fact that a catalytically inactive version of this E3 ligase, mutated in its catalytic RING finger domain, does not affect the NF-κB activation process [75]. In contrast, it has been proposed that the E3 ligase activity of TRAF2 participates in TNF-R1 signaling with sphingosine kinase 1 (Sphk1) as a co-factor [111]. However, recent studies using *Sphk1* KO cells do not support this model [112]. Nevertheless, TRAF2 has also been shown to be backed up by TRAF5, at least in MEFs, and enzymatic compensation may operate [113]. Finally, a paradoxical negative role of TRAF2 in TNF-R1 signaling has been observed in several instances. In particular, it has been reported that *Traf2*/*Traf5* KO MEFS exhibit up-regulation of NF-κB before and after TNF-α exposure [114]. Basal NF-κB activation may result from up-regulation of the non-canonical NF-κB pathway (see Section 7) but increased response to TNF-α is more difficult to explain. In any case, this suggests that while all primary players in the TNF-R1 signaling pathway might have been identified, details of their molecular functions still require further investigation.

### 5.2. The IL-1β R/TLR Signaling Pathways

Interleukin-1β receptor (IL-1βR), the receptor for inflammatory cytokine IL-1β, and toll-like receptors (TLRs), the receptors involved in the recognition of PAMPs, share similarities with regard to their mechanism of NF-κB activation [115,116]. This is due to the presence in their intracytoplasmic domain of the so-called toll-interleukin receptor (TIR) domain which interacts with a collection of related TIR-containing adaptor molecules [117]. These adaptors allow recruitment of the same set of proteins to transmit the signal that activates IKK. Nevertheless, differences exist that are mostly related to the ability of a subset of TLRs to additionally induce NF-κB from intracellular locations.

**Figure 8:**
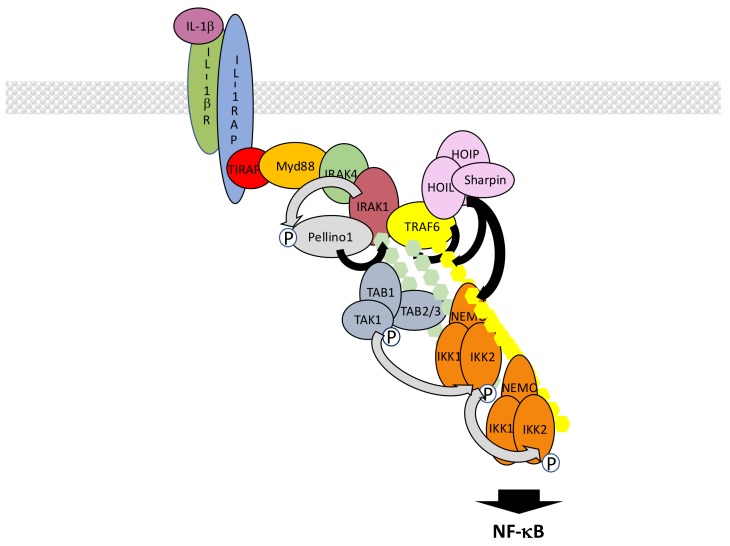
IL-1βR1 signaling pathway. Components and mechanisms ensuring signal transduction in this pathway are depicted, with black arrows indicating ubiquitination processes and grey arrows phosphorylation. M1- and K63-linked polyubiquitination is indicated with yellow and green hexagons, respectively. See text for details.

In the case of IL-1βR, interaction of a dimer composed of IL-1βR itself and IL1 receptor accessory protein (IL1RAP) with IL-1β allows the recruitment of toll-interleukin receptor adaptor protein (TIRAP) and myeloid differentiation primary response 88 (Myd88) at the TIR domain of IL-1βR, both proteins displaying TIR domains (Figure 8). The death domain (DD) of Myd88 then attracts sequentially DD-containing interleukin-1 receptor-associated kinase 4 (IRAK4), IRAK1 and IRAK2 kinases to form an oligomeric complex called the myddosome [118,119]. Through their PXEXXE motifs IRAK1 and 2 induce the recruitment and multimerization-induced activation of E3 ligase TRAF6 [120]. Concomitantly, the other E3 ligases Pellino1 and 2 interact with IRAK1 or 4 through their forkhead-associated (FHA) domain [121,122,123] and are activated by phosphorylation. Together with TRAF6, they synthesize K63-linked chains that are recognized by the TAK and IKK complex, triggering IKK activation. In this context, Pellino proteins appear to play the main role in K63-linked chain synthesis, mostly targeting IRAK1, while TRAF6 fulfills an additional scaffolding function by recruiting LUBAC. This allows full activation of NF-κB through the synthesis of M1-linked chains of ubiquitin and recruitment of IKK through NEMO. Again, K63- and M1-linked polyubiquitin chains may not be synthesized independently. Indeed, it has been shown that hybrid chains are formed and attached to IRAK1 [124]. In addition, unanchored chains are also synthesized [125] but their exact function remains uncertain.

The whole process described here is tightly time-regulated and may rely on sub-complexes with distinct intracellular locations. It has been proposed that after initiation of the activation process at the membrane a TRAF6/TAK1-containing complex translocates to the cytosol [126], with this event requiring TRAF6/c-IAP-dependent degradation of Myd88-associated TRAF3 [127].

This dependency on intracellular localization is further illustrated with data concerning signal transmission by TLR family members. In the case of TLR4, which recognizes Gram-negative bacteria-derived LPS in combination with myeloid differentiation factor 2 (MD2) and/or cluster of differentiation 14 (CD14), the signaling pathway described above for IL-1βR operates and generates a first wave of NF-κB activation. Then, TLR4 is internalized and relocated to the endosome where it generates a second wave of NF-κB activation. At this step, the TIR domain of TLR4 attracts TRIF-related adaptor molecule (TRAM) which itself binds to protein TIR domain-containing adaptor-inducing interferon-β (TRIF) [128]. TRIF-dependent signaling to NF-κB involves members of the TRAF family such as TRAF2 and TRAF6 which both interact with TRIF through TRAF binding domains [129]. Interestingly, TRIF also interacts with RIPK1 through RIP homotypic interaction motif (RHIM)/RHIM homotypic binding [130,131]. K63-linked ubiquitination of TRIF and TRAF6 is supposed to allow recruitment of TAK1 and IKK for activating IKK but molecular details are missing, especially those concerning the exact function of RIP, maybe in relation with TRAF2. Another E3 ligase, Pellino1, has to be considered since it is necessary for TRIF-dependent NF-κB activation and would participate in RIPK1 recruitment and ubiquitination [132]. Intracellular members of the TLR family recognizing a diversity of ligands such as TLR3 (ligand: double strand RNA), TLR7 (ligand: single strand RNA) and TLR9 (ligand: CpG DNA), also activate NF-κB through this TRIF-dependent pathway.

Ubiquitin-related negative regulation of the IL-1β/TLR signaling pathways occurs at several levels. At the membrane level, membrane-associated E3 ligase membrane associated RING-CH 8 (MARCH8) down regulates IL1RAP amount through K48-linked ubiquitination [133]. This results in decreased recruitment of Myd88 and IRAK-1 after IL-1β stimulation. After its binding to Myd88, phospho-activated OTUD4 is the DUB that removes K63-linked ubiquitin chains from Myd88, IRAK1 and TRAF6, decreasing IL-1βR and TLR4 signaling [134]. Among these components ubiquitinated TRAF6 represents a regulatory target for a large collection of additional proteins. Among them are USPs such as USP2a, USP4 and USP20 that digest K63-linked ubiquitin chains and inhibit IL-1β/TLR signaling [135,136,137]. CYLD, A20, Itch, small heterodimer partner (SHP) and TRAF family member-associated NF-κB activator (TANK) also negatively regulate K63-linked ubiquitination of TRAF6 through distinct mechanisms. CYLD and A20 are DUBs, the latter one working with Itch [138,139], whereas SHP and TANK are TRAF6-binding proteins indirectly affecting the TRAF6 ubiquitination status [140,141]. In the case of TANK, a TANK-monocyte chemotactic protein-induced protein-1 (MCPIP1)-USP10 complex operates, with TANK playing an adaptor role for TRAF6 deubiquitination by USP10. Finally, RNF19A, through nod-like receptor protein 11 (NLRP11), WW domain-containing protein ligase 1 (WWP1) and TRIM38 are E3 ligases that conjugate TRAF6 with K48-linked chains, causing its degradation [142,143,144].

Another component of the TLR4-dependent NF-κB activation process whose function is fine-tuned by a ubiquitin-mediated process is TRAF3 (see above). Its ubiquitination-dependent degradation is down-regulated by USP25 [145].

### 5.3. The Nod1/Nod2 Signaling Pathway

Nucleotide-binding oligomerization domain 1 (Nod1) and Nod2 are two cytoplasmic members of the NOD-LRR (leucine-rich repeat) family with CARD (caspase recruitment domain) (NLRC) that recognize bacterial peptidoglycans [146,147,148]. Nod1 detects γ-d-glutamyl-mesodiaminopimelic acid (iE-DAP) from Gram-negative bacteria and a subset of Gram-positive bacteria. Nod2 detects muramyl dipeptide (MDP) structures from both Gram-positive and Gram-negative bacteria. They both exhibit one (Nod1) or two (Nod2) CARD domains at the N-terminus, followed by a NOD domain and a series of LRRs. These repeats are responsible for ligand binding.

**Figure 9:**
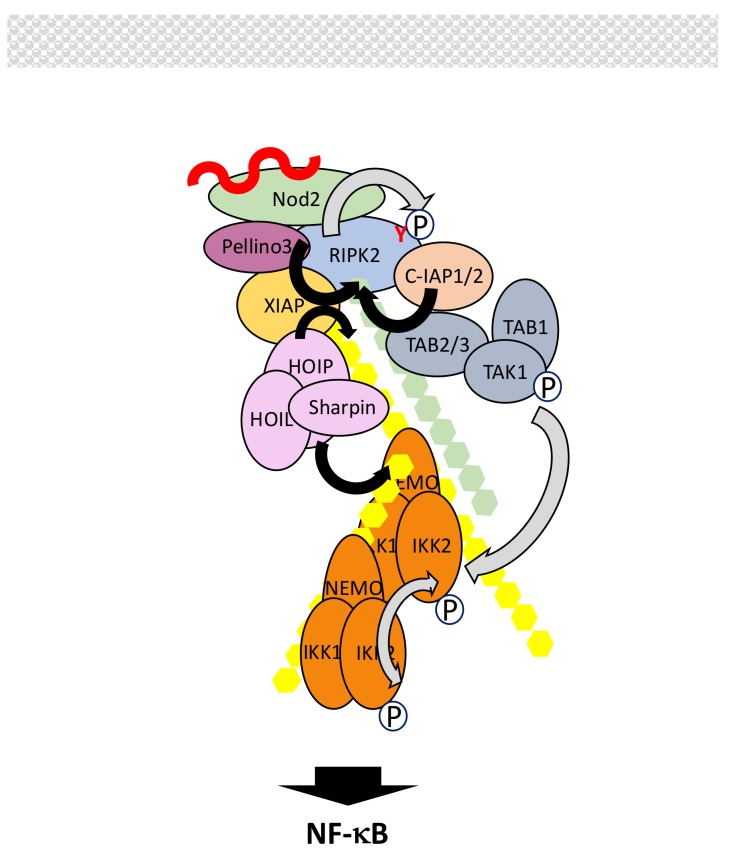
Nod1/2 signaling pathway. Components and mechanisms ensuring signal transduction in this pathway are depicted, with black arrows indicating ubiquitination processes and grey arrows phosphorylation. M1- and K63-linked polyubiquitination is indicated with yellow and green hexagons, respectively. Auto-activating Tyrosine phosphorylation of RIPK2 is indicated with a red Y. See text for details.

In resting cells, Nod proteins present an autoinhibited monomeric conformation. Upon interaction with their ligands they self-oligomerize and recruit RIPK2 through homotypic CARD-CARD interactions. This triggers a complex set of phosphorylation and ubiquitination events whose ultimate function is to allow the recruitment of IKK and its kinase TAK1 (Figure 9). Surprisingly, the tyrosine kinase activity of RIPK2, but not the serine/threonine one, operates, inducing RIPK2 autophosphorylation at residue Tyr474 [149]. Ubiquitination of RIPK2, which involves residue Lys209, also occurs upon its interaction with Nod2 [150,151]. Several distinct E3 ligases may regulate this process. Among them are c-IAPs, X-linked inhibitor of apoptosis protein (XIAP), Pellino3 and TRAFs such as TRAF2, TRAF5 and TRAF6. C-IAPS have been shown to interact with RIPK2 and to induce its K63- and K48-linked ubiquination [152]. XIAP has also been reported to interact with the kinase domain of RIPK2 through its baculovirus inhibitor of apoptosis protein repeat 2 (Bir2) domain but it is not clear which kind of polyubiquitin chains it adds to RIPK2 [153]. Pellino is a third E3 ligase that interacts with RIPK2, through its FHA domain, and triggers its K63-linked polyubiquitination [154]. All these ligases appear necessary for inducing NF-κB in response to Nod2 activation, so it is likely that they perform distinct functions. C-IAP/RIPK2 and Pellino3/RIPK2 interactions are correlated to K63-linked ubiquitination of RIPK2 and this may induce the recruitment of the TAK complex. Moreover, it has been shown that c-IAP2 promotes RIPK2 tyrosine phosphorylation. In contrast, XIAP may play a scaffold role to attract both the TAK complex though direct interaction with its Bir1 domain and, more importantly, the E3 LUBAC complex [155,156]. As a consequence, LUBAC would synthesize M1-linked chains, allowing the recruitment of IKK through NEMO. As in other signaling pathways presented above, induced proximity of TAK and IKK complexes would result in IKK activation. Again, hybrid ubiquitin chains may represent the genuine anchor during this process [77].

The role of TRAFs in Nod2 signaling is more controversial. It was originally reported that TRAF6 was essential for NF-κB activation [157] but this has not been confirmed by other studies. In particular, Hasegawa et al. [151] reported that instead of TRAF6, the other two TRAFs which often work jointly, TRAF2 and TRAF5 (see Section 5.1), were the active ligases for RIPK2. Considering what is said above about the other E3 targeting RIPK2, such observation requires further investigation.

Fine-tuning of Nod2 signaling and shut-off is achieved through several mechanisms. First, like Nod2, RIPK2 is kept in check in the cytoplasm before cell stimulation though its interaction with mitogen-activated protein/ extracellular signal-regulated kinase (ERK) kinase 4 (MEKK4) [158]. Upon Nod2 activation RIPK2 dissociates from MEKK4 to interact with Nod2. Second, modulation of activity and stability of several components are regulated by complex ubiquitination processes involving E3 ligases and DUBs. As described in other signaling pathways, OTULIN, through its recruitment by LUBAC, can hydrolyze M1-linked chains of ubiquitin to stop signal transmission [93]. LUBAC also provides a platform for recruiting SPATA2/CYLD with the same outcome [159,160]. Moreover, E3 ligase Itch acts as an inhibitor of Nod2 signaling though ubiquitination and inactivation of RIPK2 [161]. Interestingly, effective function of Itch requires RIPK2 tyrosine-phosphorylation. This suggests a dual positive and negative function of tyrosinated RIPK2: after allowing Nod2 signaling through a still unclear mechanism it may participate in its shut-off. Since A20 has been shown to negatively regulate Nod2/RIPK2 activity through RIPK2 deubiquitination [159,162] an A20/Itch complex may be at play, as in the TNF-R1 signaling pathway (see Section 5.1). Another E3 ligase, zinc and ring finger 4 (ZNRF4), also acts at the level of RIPK2 by controlling its level through K48-linked polyubiquination [163]. The stability of active Nod2 is itself regulated by TRIM27, which conjugates Nod2 with K48-linked polyubiquitinated chains for degradation by the proteasome [164]. Finally, the SH2-containing inositol phosphatase (SHIP) negatively regulates the Nod2/NF-κB signaling pathway by impairing interaction between XIAP and RIPK2 [165].

### 5.4. The MAVS Pathway

Intracellular retinoic-inducible gene-I (RIG-I)-like receptors (RLRs) regulate the synthesis of type-I interferons (IFNs) following virus-derived RNA recognition [166,167,168]. The RLR family includes RIG-I, melanoma differentiation-associated gene 5 (MDA5) and Laboratory of Genetics and Physiology 2 (LGP2), which all bind dsRNA through their death effector domain (DEAD)/H-box RNA helicase domain. Through their CARD, RIG-I and MDA5 then activate mitochondrial antiviral-signaling protein (MAVS), which in turn activates both the NF-κB and IRF signaling pathways to trigger IFNs production. LGP2, which is devoid of CARD, was originally thought to be a negative regulator of this process but may instead facilitate viral RNA recognition by RIG-I and MDA5 through its ATPase domain [169].

**Figure 10:**
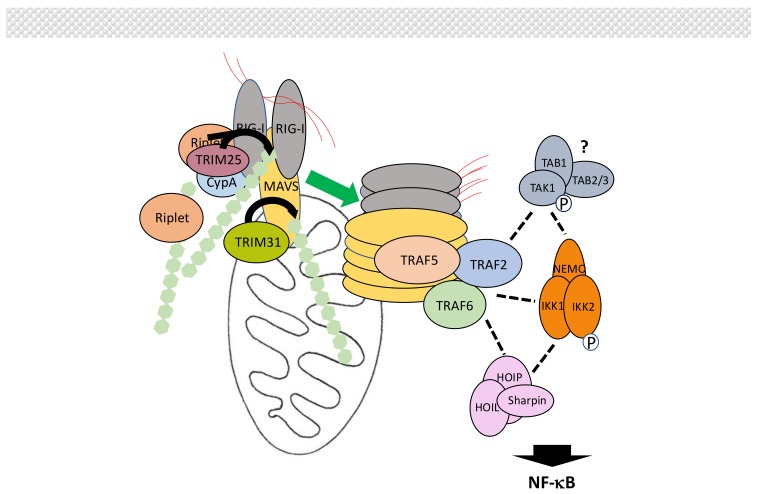
RIG-I/MAVS signaling pathway. Components and mechanisms ensuring signal transduction in this pathway are depicted, with black arrows indicating ubiquitination processes. K63-linked polyubiquitination is indicated with green hexagons. Components shown to be (formally or putatively (question mark)) required in this pathway but whose exact relationship is not defined are shown connected by broken lines. Red filaments represent activating double strand RNA. Activation occurs at the surface of the mitochondria. E3 ligases TRIM4 and MEX3C may also participate in RIG-I activating ubiquitination. See text for details.

Not surprisingly, several ubiquitination events regulate MAVS-dependent signal transduction [170,171] (Figure 10). First, upon recognition of viral RNA exposure of CARD domains induces K63-linked polyubiquitination of RIG-I at Lys172 by TRIM25 [172], with the help of cyclophilin A (CypA) [173]. This induces formation of high order RIG-I oligomers through the CARDs of RIG-I that exhibit affinity for K63-linked chains [174]. Unanchored K63-linked chains may also participate in this process [175]. Other E3 ligases have been shown to also induce K63-linked polyubiquitination of RIG-I. Riplet could help opening RIG-I and facilitate its ubiquitination by TRIM25 [176]. Following the additional identification of TRIM4 and MEX-3 homolog C (C. elegans) (MEX3C) as E3 ligase for RIG-I [177,178] Sun et al. [179] have proposed a hierarchical model of RIG-I activation by K63-linked ubiquitination. The same process of induced activation by K63-linked polyubiquitination occurs with MDA5. In this case, TRIM65 represent the active E3 ligase [180]. Then, oligomerized RIG-I and MDA5 trigger extensive polymerization of MAVS at the mitochondria surface through a prion-like mechanism [181], allowing recruitment of proteins participating in NF-κB and/or IRF activation.

We will focus here on the proteins involved in NF-κB activation, neglecting both their additional function in IRF activation and the proteins specifically regulating this parallel process. Among these participants are primarily members of the TRAF family. Indeed, MAVS harbors binding motifs for TRAF2, TRAF3, TRAF5 and TRAF6. Mutations of all these motifs is required to completely abolish NF-κB activation, with TRAF6, TRAF2 and TRAF5 performing redundant functions [182,183,184] whereas the role of TRAF3 is still controversial. The E3 activity of TRAFs is required and K63-linked polyubiquitination is necessary for the recruitment of IKK through NEMO but the exact modified partner(s) is(are) unknown. Indeed, a mutated TRAF6 that cannot be ubiquitinated remains active in this pathway. Moreover, although TRAF2 is found associated with NEMO, inhibiting its ubiquitination does not affect the NF-κB activation process either. Nevertheless, when the same inhibition is combined with a loss of HOIP expression, NF-κB activity is not induced [182] suggesting another layer of redundancy involving M1-linked chains. The participation of TAK1, which often plays the role of IKK activating kinase, is still uncertain, leaving the question open for the final step in IKK activation and its putative similarities to those described for other signaling pathways. It is worth noting that a MAVS/TRAF2/TAK1 pathway controlling MAPK p38 activation has been reported but its role in NF-κB activation has not been analyzed [185].

Modulation of MAVS activation by ubiquitin-related PTMs is complex [170]. As outlined above, TRIM25 induces K63-linked polyubiquitination of RIG-I for initiating signaling. The amount of TRIM25 is itself controlled by the DUB USP15, which removes K48-linked chains [186]. At this level, LUBAC has been proposed to also act through an unorthodox dual mechanism. It could induce TRIM25 degradation through proteasome degradation and compete with TRIM25 for RIG-I recognition [187]. Activating K63-linked chains of RIG-I are proteolyzed by USP3, USP21 and CYLD [188,189,190,191], whereas RNF122 and RNF125 can induce RIG-1 degradation through K48-linked polyubiquitination [192,193,194]. This process is inhibited by USP4 [195].

RNF125 has a broad impact on the MAVS signaling pathway since it can also act on MDA5 and MAVS to induce their degradation [193,194]. It is not the only E3 ligase inducing MAVS degradation by the proteasome. The HEC domain containing E3 ligases Smad ubiquitin regulatory factor 1 (Smurf1), through Nedd4 family interacting protein 1 (Ndfip1), [196] and Smurf2 [197] inhibit interferon induction by promoting K48-linked polyubiquitination of MAVS. MARCH5 binds to MAVS, only after its polymerization, to induce its degradation [198]. RNF5 also acts after signal induction [199]. Activity of these two ligases may be controlled by inactive Rhomboid 2 (iRhom2) [200]. Finally, after its induced synthesis following viral infection, poly(RC) binding protein 2 (PCBP2) negatively regulates the RIG-I/MAVS signaling pathway by interacting with MAVS and recruiting E3 ligase atrophin-1-interacting protein 4 (AIP4) for MAVS degradation by the proteasome [201].

### 5.5. The cGAS/STING Pathway

**Figure 11:**
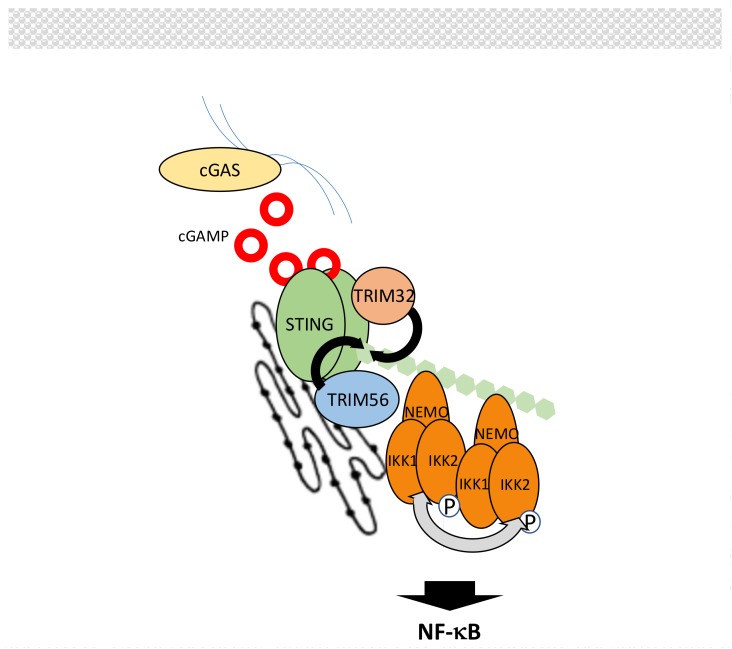
cGAS/STING signaling pathway. Components and mechanisms ensuring signal transduction in this pathway are depicted, with black arrows indicating ubiquitination processes and grey arrows phosphorylation. K63-linked polyubiquitination is indicated with green hexagons. Blue filaments represent activating double strand DNA. Activation occurs at the surface of the endoplasmic reticulum. See text for details.

B-form DNA originating from invading/replicating pathogens or self-DNA causing auto-immune response is sensed in the cytoplasm by cyclic guanosine monophosphate (GMP)-adenosine monophosphate (AMP) synthase (cGAS) [202,203,204]. This induces the production of 2′-5′-cyclic guanosine adenosine monophosphate (2′-5′-cGAMP), an atypical cyclic dinucleotide second messenger that is recognized by endoplasmic reticulum (ER) membrane-associated protein stimulator of interferon genes (STING) [205] (Figure 11). Then, STING activates the NF-κB and IRF3 pathways to induce interferon production.

Details of NF-κB activation by STING are lacking but, as for other NF-κB pathways, this process is heavily regulated by ubiquitination. First, E3 ligases TRIM32 and TRIM56 promote K63-linked ubiquitination of STING to induce NF-κB signaling [206,207], most likely by recruitment of IKK through NEMO [206]. This step is negatively controlled by the DUB USP13 [208]. Second, the stability of STING is controlled by E3 ligases RNF5 and TRIM30α (murine specific) that target STING for degradation through K48-linked polyubiquitination [209,210]. This degradation is counteracted by E3 ligases USP18 and USP20 that deconjugate K48-linked chains from STING [211]. Through a distinct mechanism, involving SUMOylation, TRIM38 also interfere with K48 ubiquitination of STING [212].

Upstream of STING, cGAS activity is also regulated by ubiquitination. ER-associated E3 ligase RNF185 has been shown to induce K27-linked polyubiquitination of cGAS and to increase its activity [213]. Moreover, degradation of cGAS by K48-linked chains, carried out by an unknown E3 ligase, is inhibited by TRIM14 working in concert with USP14 [214].

### 5.6. The TCR/BCR Pathway

**Figure 12:**
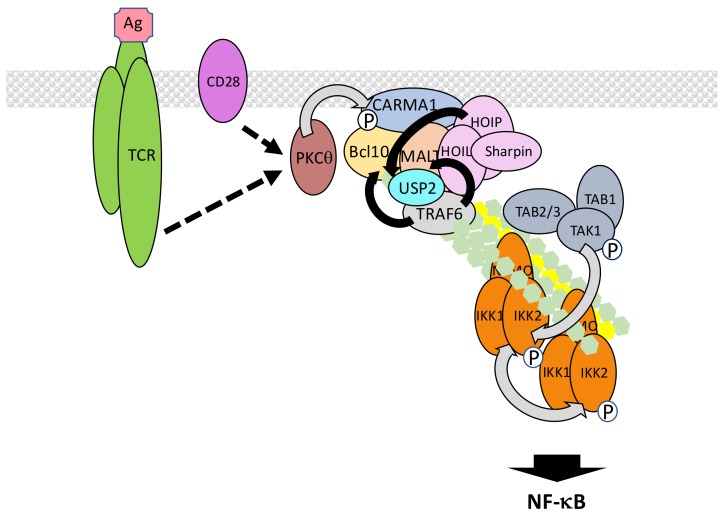
TCR signaling pathway. Components and mechanisms ensuring signal transduction in this pathway are depicted, with black arrows indicating ubiquitination processes and grey arrows phosphorylation. M1- and K63-linked polyubiquitination is indicated with yellow and green hexagons, respectively. Events occurring upstream of PKC activation are not shown and indicated by broken arrows. See text for details.

The T cell receptor (TCR) and B cell receptor (BCR) expressed on lymphocytes recognize foreign antigens and represent the pillars of adaptive immunity by allowing the mounting of the memory response. Both trigger NF-κB activation through similar components and processes. Proximal signaling at the TCR/BCR [215] will not be detailed here. We will only concentrate on the shared events following PKCθ and PKCβ activation in T cells and B cells, respectively [216,217] (Figure 12). These kinases both targets CARD-containing a membrane-associated guanylate kinase (MAGUK) protein 1 (CARMA1), a member of a small family of adaptors displaying a MAGUK domain. In resting lymphocytes CARMA1 adopts a closed inactive conformation associated with the plasma membrane. Upon phosphorylation by PKCθ/β, CARMA1 opens and oligomerizes through its CC domains [218]. This provides a raft-associated platform for attracting through CARD/CARD interaction B cell lymphoma 10 (Bcl10), which is bound to mucosa-associated lymphoid tissue (MALT) lymphoma associated translocation protein 1 (MALT1). This ternary complex is called the CARMA1/Bcl10/MALT1 (CBM) complex. Through its TRAF6 binding motifs MALT1 then recruits TRAF6, which adds K63-linked polyubiquitin chains on several components of the CBM complex, among them MALT1 and Bcl10 [219]. MALT1/TRAF6 interaction may be reinforced by co-interaction with USP2a [220]. The resulting cluster of ubiquitinated proteins finally attracts the TAK and IKK complexes for NF-κB activation. This last step may occur in the cytosol [221,222].

This basic model can be further refined taking into account the following observations. First, the E3 ligase involved in K63-linked CBM ubiquitination/IKK activation may not be uniquely TRAF6 since NF-κB activation still occurs in *Traf6* KO T lymphocytes [223]. TRAF2 could play redundant functions with TRAF6 to ensure CBM ubiquitination [219]. Alternatively, the E3 ligase mind bomb 2 (MIB2) may also be a candidate since it interacts with Bcl10 and induces K63-linked polyubiquitination of CBM components [224], Second, M1-linked chains of ubiquitin may also regulate TCR/BCR signaling. Nevertheless, the published data is controversial, even considering putative different requirements during TCR versus BCR signaling. Indeed, whereas LUBAC is necessary for TCR signaling, the catalytic activity of HOIP subunit is not required [225], suggesting a scaffold function of LUBAC maybe in relation with TRAF6. However, M1-linked polyubiquitination of Bcl10 has been observed following TCR stimulation [226] and during chronic B cell activation [227] suggesting the specific requirement of LUBAC-dependent linkage of M1 chains. A possible explanation for the observations of Dubois et al. [225] would be that quantitative evaluation of TCR/BCR signal transduction upon abolished E3 ligase activity of LUBAC is complicated by the remaining K63-linked dependent activation. Finally, the identity of the kinase activating IKK is not firmly defined in this particular pathway. TAK1 represents an obvious candidate, given its broad involvement in NF-κB-inducing pathways, but it is dispensable in some situations [228]. Another MEKK, MEKK3, may fulfill the same function in parallel or redundantly [229].

Several negative regulators of ubiquitin-related processes in TCR/BCR signaling have been identified. A20 hydrolyzes K63-linked chains on MALT1 to shut-off NF-κB activation [230]. This function is itself counteracted by MALT1, a member of the paracaspase family able to cleave and inactivate A20 [231]. MALT1 also acts the same way on CYLD, which deubiquitinates TAK1 [232], and possibly TRAF6 and NEMO during TCR signaling. In this case, the cleavage of CYLD does not affect the amplitude of NF-κB signaling but participates in JNK activation [233]. Finally, MALT1 cleaves HOIL-1 to generate a dominant-negative version of LUBAC [234]. Therefore, a key function of MALT1 is to control the amplitude of TCR/BCR signaling, by regulating LUBAC and A20 levels, after the activating signal has been transmitted.

Another DUB that down-regulates NF-κB activation upon TCR stimulation is USP34 which acts at a step downstream of IKK activation [235]. It may regulate IκBα degradation. How, and to what extent, USP34 is specifically activated by TCR stimulation still requires further investigation.

Finally, the ubiquitin-associated and SH3 domain containing 3A (UBASH3A) is an intriguing negative regulator of TCR signaling to NF-κB. UBASH3A down-regulates IKK activation and Ge et al. [236] have proposed that it may work by binding to TAK1 and NEMO, therefore hindering their recognition of K63-linked chains. Very interestingly, the gene coding for this ubiquitin-binding protein is located at a type 1 diabetes risk locus. In addition, genetic variants of *UBASH3A* are associated with several other autoimmune diseases.

### 5.7. The Genotoxic Stress Pathway

DNA damaging agents, such as ionizing radiation or chemotherapeutic drugs, induce a genotoxic stress that triggers NF-κB activation, resulting in cell protection from death [237,238]. Understanding the molecular details of this process is therefore of the utmost importance for improving the efficiency of several cancer treatments. 

**Figure 13:**
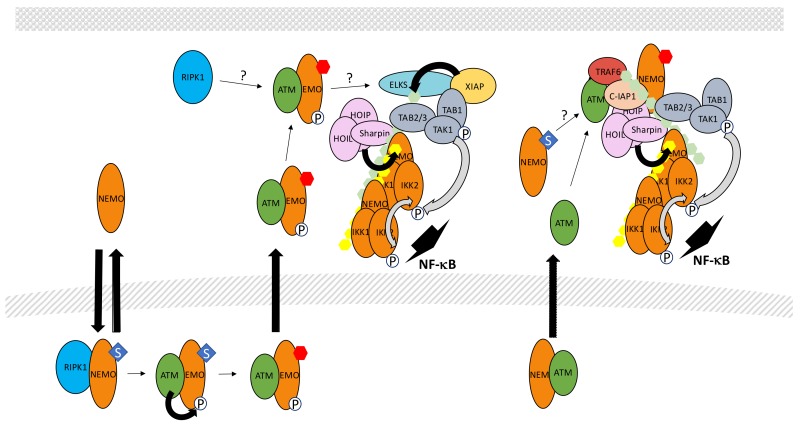
Genotoxic stress signaling pathway. Components and mechanisms ensuring signal transduction in the two proposed pathways are depicted, with black arrows indicating ubiquitination processes and grey arrows phosphorylation. M1- and K63-linked polyubiquitination is indicated with yellow and green hexagons, respectively. Blue squares labeled S indicate sumoylation whereas red hexagons indicate monoubiquitination. The participation of RIPK1 in the cytoplasmic events is indicated but with a question mark since how it relates to these two pathways, or another one, is unclear. See text for details.

In this specific situation, unusual PTMs of NEMO have been discovered to play critical functions in NF-κB activation through IKK. More specifically, a pool of free NEMO exists in resting cells and can shuttle between the cytoplasm and the nucleus (Figure 13). Miyamoto et al. [239] were the first to show that NEMO sumoylation modulates this shuttling upon DNA damage, causing nuclear accumulation of NEMO. SUMO conjugation occurs at Lys277/Lys 309 and protein inhibitor of activated STAT (signal transducer and activator of transcription) y (PIASy) is the SUMO E3 ligase involved in this process [240]. Interestingly, the binding site of PIASy on NEMO overlaps with its IKK binding site confirming that sumoylation acts on a free pool of NEMO.

In the nucleus, sumoylated NEMO has been proposed to interact with P53-induced protein with a death domain (PIDD) and RIPK1 [241] but the requirement for PIDD in the sumoylation process is controversial [242]. Alternatively, PolyADP-ribose polymerase 1 (PARP-1) may provide a link between DNA damage sensing and NEMO sumoylation. It has been shown that PolyADP-ribose-modified PARP-1 triggers the formation of a complex containing NEMO, PIASy and ataxia telangiectasia mutated (ATM), a kinase responding to DNA double strand breaks [243]. Nevertheless, *Parp* KO mouse embryonic fibroblasts have been shown to properly activate NF-κB upon exposure to DNA damaging agents. Therefore, the identity of the proteins regulating sumoylation of NEMO in the nucleus remains uncertain. In contrast, the role of ATM in signal transmission is firmly established. Indeed, it regulates a NEMO-dependent activation process of NF-κB following DNA damage [244]. Moreover, the same DNA damaging treatment induces NEMO phosphorylation by ATM at Ser85 [245]. Phosphorylation of NEMO is not required for its sumoylation but instead, controls its monoubiquitination at Lys277 and Lys309. The identity of the E3 ligase involved in this monoubiquitination and how sumoylated sites are converted to monoubiquitinated sites remains unclear. In any case, this PTM results in the nuclear export of NEMO accompanied with a fraction of ATM.

How NEMO/ATM then participates in IKK activation in the cytoplasm is still a matter of debate. Clearly, the TAK1 complex is involved in this process since *Tak1* KO cells cannot activate NF-κB upon DNA damage. The complex NEMO/ATM may induce K63-linked ubiquitination of protein rich in amino acids E, L, K and S (ELKS) by E3 ligase XIAP through a mechanism that is still undefined [246]. This would then result in the recruitment and activation of TAK1. At the same time, the IKK complex would also be recruited and activated by TAK1, similarly to what has been described in other signaling pathways (see above). Additional proteins participate in this process. First, RIPK1, which plays a function in the nucleus events leading to NEMO modification, has been shown to also translocate in the cytoplasm, associated with NEMO and ATM, and is required for TAK complex recruitment [247]. Second, LUBAC also participates in the genotoxic stress pathway and modifies NEMO with M1-linked chains [248]. Surprisingly, in the Tergaonkar/Miyamoto study (246) the NUB domain of NEMO was shown to be dispensable for NF-κB activation upon DNA damage, something quite difficult to reconcile with a critical involvement of M1-linked and K63-linked polyubiquitin chains in TAK1-dependent NF-κB activation.

Alternatively, Hinz et al. [249] proposed that upon cytoplasmic release ATM interacts with TRAF6 through a TRAF6-binding domain and form a complex with c-IAP1 and NEMO to activate IKK through a “standard” TAK1-dependent mode. Importantly, monoubiquitination of NEMO was also observed in this study. It occurred after sumoylation, as reported by Wu et al. [245], although, in this case, exclusively in the cytoplasm. Moreover, monoubiquitination was observed only after recruitment of NEMO to ATM/TRAF6/c-IAP1 and required the NEMO NUB domain. The discrepancies between these studies and the relationship between these different complexes, if indeed they represent distinct entities, require further characterization. Interestingly, Jin et al. [250] demonstrated a non-redundant function of cIAPs and XIAP in the genotoxic stress pathway and proposed that c-IAP1 may be the E3 ligase responsible for NEMO monoubiquitination. This would explain the sequence of events reported by Hinz et al. [249] and further demonstrates the high versatility of c-IAP1, also able to induce 48-, K63-and, possibly, K11-linked polyubiquitination.

Negative regulation of the genotoxic stress pathway at the level of NF-κB induction is incompletely characterized. The NEMO sumoylation step is down-regulated by desumoylase SUMO-specific protease 2 (SENP2) [251]. Regarding the ubiquitin-related modifications, USP10 has been shown to decrease M1-linked ubiquitination of NEMO during DNA damage induction [252]. USP10 requires NF-κB-induced MCPIP1 for binding to NEMO, suggesting that MCPIP1 regulates a negative feedback mechanism. Interestingly, the same authors have shown that USP10 may also target ubiquitinated TRAF6 upon genotoxic stress through the TANK-MCPIP1-USP10 complex described above (see Section 5.2) [141].

## 6. Regulated Ubiquitination of TAK1 and IKK Complexes

In the above section we examined various signaling pathways and described the ubiquitin-related post-translational modifications of their specific components. Activation of these pathways eventually results in the recruitment of identical proteins that channel the signal towards NF-κB. Among them are the subunits of the TAK1 and IKK complexes, members of the TRAF family and core components of the NF-κB system (NF-κB subunits and IκBs). Here, we will describe how these core actors of NF-κB activation are modified through ubiquitination, affecting their function to impact on signaling. The broad versus specific involvement of these modifications is often not analyzed so we will only mention the reported associated pathways without further extrapolations to others.

### 6.1. Regulated Ubiquitination of TAK1 Complex Components

Not surprisingly, ubiquitination of the various components of the TAK1 complex regulates not only their activity but also their stability, impacting on NF-κB induction [253]. Following TNF-α and IL-1β exposure, K63-linked polyubiquitination of TAK1 occurs mainly at Lys158 but also possibly at Lys34, Lys209 and Lys562 [254,255]. TRAF2 and TRAF6 may be the respective E3 ligases involved in TAK1 ubiquitination but an alternative candidate is the TAK1-binding protein TRIM8 [256]. Ubiquitination of TAK1 could help to consolidate its interaction with the IKK complex through NEMO for full activation. As mentioned above, CYLD may negatively control this process and work with Itch as a partner to secondarily induce TAK1 degradation through K48-linked ubiquitination. Other deubiquitinases targeting TAK1 are USP4 and USP18. USP4, in particular, downregulates NF-κB activation by TNF-α and IL-1β [257] while USP18 was originally described as targeting TAK1 in the TCR pathway [258], it is also involved in TLR signaling [259]. Finally, Pellino3b negatively regulates TAK1-dependent NF-κB activation by IL-1β [260].

In a specific situation, during maternal-to-zygotic transition, E3 ligase RNF114 induces TAB1 degradation through K48-linked polyubiquitination [261]. Surprisingly, this results in NF-κB activation through a poorly defined mechanism. K63-linked polyubiquitination of TAB1 at several Lys residues has also been reported [262] but its relevance in NF-κB signaling has not been demonstrated. The same applies to Itch-dependent TAB1 degradation [263].

TAB2/3 stability has been shown to be controlled by at least three distinct E3 ligases. The first one, TRIM38, interacts with TAB2/3 in the TNF-α and IL-1β signaling pathways and induces their degradation by the lysosome through an E3 ligase-independent process [264]. TLR-induced TRIM30α appears to act the same way [265]. Finally, RNF4, also targets TAB2 for degradation by the lysosome pathways but, in this specific case, its E3 ligase activity is required [266]. This peculiar mode of disposal and its exact association with ubiquitination processes deserves additional investigation.

### 6.2. Regulated Ubiquitination of IKK Complex Components

As discussed above, the main function of NEMO is to recognize polyubiquitin chains, but it can also be modified by ubiquitination. Zhou et al. [267] were the first to identify a Bcl10-dependent site of ubiquitination on NEMO, located in the zinc finger (Lys399) and participating in NF-κB activation by the TCR. It is unlikely that Bcl10 is the E3 ligase involved in this pathway as originally proposed. Subsequently, Lys285 was identified as modified during Nod2 signaling [268]. In this situation, TRAF6 was proposed to be the required E3 ligase although its participation in Nod1/Nod2 signaling remains controversial (see Section 5.3). Finally, several residues targeted by TRAF6 have been reported to be affected by a *NEMO* mutation causing incontinentia pigmenti pathology (see below). In all these cases, the identified lysine residues are believed to be specifically modified by K63-linked chains.

More recently, M1-linked polyubiquitination has been shown to play a critical function in NF-κB activation, as explained above. This process involves LUBAC and its first identified target was NEMO. Lys285 and Lys309, which are located within the NUB domain represent preferentially modified residues [269].

The effective participation of these different ubiquitination sites of NEMO in general or pathway-specific NF-κB activation has been investigated in vivo. Mutating only Lys399 in mice does not generate a severe phenotype, notably at the T cell level, but a reduced response of macrophages to LPS is observed, which is associated with resistance to endotoxic shock [270]. Mutating both Lys 285 and 399 residues results in strong impairment of NF-κB activation, and early TNF-dependent male lethality, similar to the one seen in *Nemo* KO mice [271,272]. In addition, the same mice rescued with a *Tnfr1* KO display an impaired response of macrophages to Nod2, LPS or IL-1β [271]. Thus, NEMO ubiquitination plays an important and broad role in NF-κB signaling. Although originally identified individually, the various ubiquitinated Lys residues may fulfil a similar function, alone or in combination, i.e., to help reinforce, through K63- and M1-linked chains, interactions between TAK1 and IKK complexes for optimal IKK activation.

Other modifications of NEMO through ubiquitination have been reported. They can positively or negatively affect its function. First, as described in Section 5.7, NEMO can be monoubiquitinated in the nucleus following DNA damage and this is a key activating event. Second, TRIM23 has been shown to ubiquitinate NEMO, through K27-linked chains, in the RIG-I/MAVS pathway [273]. The precise function of this peculiar kind of modification remains unknown but it is required for NF-κB activation. Regarding the negative regulation of NEMO function by ubiquitination, Zotti et al. [274] have shown that atypical TRAF protein TRAF7 is a NEMO interactor able to induce its Lys29-linked polyubiquitination for degradation by the lysosome. This results in impaired NF-κB activation by TNF-α. In another setting, myogenic differentiation, TRAF7/NEMO interaction and NEMO ubiquitination would instead positively regulate NF-κB [275].

Other modes of NEMO ubiquitination modulation have been shown to control its activity and down-regulate NF-κB activation. For instance, USP18, already known to inhibit TLR signaling by deubiquitinating TAK1, as mentioned in Section 6.1, also acts on NEMO [259]. EGL nine homolog 3 (EGLN3), a member of a family of prolyl hydroxylase, may negatively regulate NF-κB signaling by inhibiting NEMO ubiquitination by c-IAP1 [276]. TRIM13, an ER resident E3 ligase, interacts with NEMO and can also induce its deubiquitination through an unknown process [277]. Finally, HSCARG has been proposed to negatively regulate TNF-α-induced NF-κB activation by interacting with NEMO and inducing its deubiquitination through the recruitment of USP7 [278].

In contrast to the NEMO regulatory subunit, little is known regarding ubiquitin-regulated modification of the IKK catalytic subunits. An intriguing observation made by Niida et al. [279] suggests that TRIM21 may monoubiquitinate activated IKK2 to induce its disposal by autophagosomes.

## 7. Regulated Ubiquitination in the Non-Canonical Pathway of NF-κB Activation

As mentioned above, NIK is constitutively degraded in resting cells and stabilized upon stimulation, inducing IKK1 activity. This critical switch is regulated by intricate ubiquitin-dependent events. First, basal degradation of NIK is dependent on E3 ligase TRAF3: newly synthesized NIK associates with TRAF3 and is ubiquitinated with K48-linked chains, inducing its degradation by the proteasome [280]. Second, upon cell stimulation, specific receptors such as the CD40 or B-cell activating factor receptor (BAFF-R) recruit TRAF3 for degradation. This results in NIK accumulation and activation of the non-canonical pathway.

This basic model has been substantially refined. Although TRAF3 is essential in controlling the amount of NIK, it does not work alone but within a multimolecular E3 complex also including TRAF2 and cIAP1/2 [281,282,283]. In this complex TRAF3 is not the genuine NIK E3 ligase but plays an adaptor role for recruiting TRAF2, which itself contains a cIAP binding site. As a consequence, cIAP1/2 bound to TRAF2 acts on NIK to induce its degradation in resting cells. In this situation, cIAP1 and c-IAP2 appear functionally redundant.

How this degradative process is interrupted following stimulation is not fully understood. It has been shown that when CD40 or BAFFR bind their ligands TRAF2 and TRAF3 are recruited to the plasma membrane lipid-raft compartment [284,285]. This recruitment could initiate TRAF2-mediated polyubiquitination of both TRAFs and their subsequent proteasome-mediated degradation, causing NIK accumulation. Alternatively, c-IAP1/2 may be the active player at this level also. In this case, c-IAP1/2 catalytic activity may be augmented by TRAF2-dependent K63 ubiquitination to fulfil this specific function [281,286]. TRAF3 ubiquitination following stimulation is also supposed to help recruiting OTUD7B, an OTU domain-containing DUB, which limits TRAF3 degradation [287]. Consequently, lack of OTUD7B results in hyperactivation of the non-canonical NF-κB pathway.

Notably, STING also has the ability to activate the non-canonical NF-κB pathway through TRAF3, but the molecular mechanism involved is unknown. This deserves closer examination given the recently identified importance of the non-canonical activation of NF-κB via STING in chromosomal instability-driven metastasis [288].

## 8. Regulated Ubiquitination of NF-κB Proteins

DNA transcription involves complex machinery involving core components for RNA synthesis, transcription factors and co-regulators. Ubiquitination regulates the activity of all these elements, including those participating in NF-κB-dependent gene transcription. Sacani et al. [289] in particular were the first to demonstrate that the promoter bound p50/RelA can be degraded by the proteasome for terminating the NF-κB signaling. Thus, in situ degradation of NF-κB appears as an important down-regulation mechanism in addition to the re-synthesis of IκB and its cytoplasm/nucleus shuttling which participates in NF-κB-dependent transcription shut-off by dissociating NF-κB dimers from DNA.

Over the years, several E3 ligases have been identified as targeting the RelA subunit and ensuring the proper level and timing of gene expression. They all conjugate K48-linked chains of ubiquitin to RelA, inducing its degradation by the nuclear proteasome. Among them are copper metabolism MURR1 domain-containing 1 (COMMD1), PDZ and LIM domain 2 (PDLIM2), peroxisome proliferator-activated receptor (PPARγ) and CHIP. COMMD1 induces RelA degradation by recruiting an E3 complex including Elongins B/C, Cul2 and the suppressor of cytokine signaling 1 (SOCS1) [290]. PDLIM2 binds RelA in the nucleus and promotes its ubiquitination at discrete intranuclear compartments [291]. Interestingly, another member of the LIM family, PDLIM1, inhibits NF-κB signaling by a different mechanism, i.e., by the sequestration of RelA in the cytoplasm [292]. Moreover, PPARγ shuts off NF-κB signaling by binding to RelA and inducing through Lys28 its K48-linked ubiquitination and degradation [293]. Finally, CHIP can induce the ubiquitination and degradation of several tumour related proteins, including RelA [294]. So far, the only identified proteins limiting the RelA degradative processes are the nuclear DUB USP48, which works in concert with the COP9 signalosome to stabilize RelA through removal of K48-linked chains [295], and USP7, which interacts with DNA-bound RelA and increases its residency time at promoters by antagonizing degradative ubiquitination [296].

Monoubiquitination of RelA also occurs in the nucleus and negatively impacts on its transcriptional activity, in particular by interfering with its ability to interact with its co-activator CBP [297]. Since monoubiquitination of RelA has been observed on a mutated form of the protein, i.e., upon mutations of its phospho-acceptor sites, or following proteasome inhibition, the physiological relevance of this observation has yet to be firmly established.

Nuclear p50 can also be modified by ubiquitination. As mentioned above, p50 does not contain any TAD, but upon association with co-transcription activator Bcl3, participates in the positive regulation of transcription either with RelA, as a p50/RelA heterodimer, or as a p50/p50 homodimer. In contrast, formation of p50 dimers in the absence of Bcl3 results in negative regulation of transcription. This situation also induces p50 destabilization by K48-linked polyubiquitination [298]. How Bcl3 protects p50 from degradation remains undefined.

Transcriptional activity of c-Rel subunit is also regulated by degradative ubiquitination. In T cells, c-Rel is ubiquitinated upon TCR stimulation by Pelino1, which in this setting, promotes K48-linked polyubiquitination of c-Rel and its proteasome-dependent degradation [299]. Since Pellino1 is mostly known to act through K63-linked polyubiquitination (see Section 5.2) how it regulates K48-linked polyubiquitin chain formation remains unclear. This degradation process impacts mostly on late-phase NF-κB activation, suggesting a specific effect on c-Rel-only containing NF-κB dimers. It is unknown if this mode of c-Rel regulation is stimulus specific. This might be the case since in another cellular setting, i.e., macrophages stimulated by LPS, c-Rel stability appears to be controlled by a distinct mechanism involving TRAF2 in combination with TRAF3 and c-IAP1 [300,301]. Molecular details are lacking but this TRAF2-driven degradation process of c-Rel limits the expression of proinflammatory cytokines.

Finally, RelB can be modified by ubiquitination and this affects not only its stability but also its activity. Indeed, nuclear ubiquitination of RelB, other than K63- or 48-linked polyubiquitination, is required for transcription [302]. Stimulus-dependent degradation of RelB can also occur [303]. Finally, sumoylation has also been reported to down-regulate RelB transcriptional activity [304].

## 9. In Vivo Relevance of Ubiquitin-Dependent NF-κB Processes

NF-κB-related ubiquitination/ubiquitin recognition processes described above at the protein level, regulate many important cellular/organismal functions impacting on human health. Indeed, several inherited pathologies recently identified are due to mutations on proteins involved in NF-κB signaling that impair ubiquitin-related processes [305]. Not surprisingly, given the close relationship existing between NF-κB and receptors participating in innate and acquired immunity, these diseases are associated with immunodeficiency and/or deregulated inflammation.

### 9.1. NEMO Mutations

In humans, *NEMO* mutations can cause two distinct pathologies [306]. Loss-of-function mutations of *NEMO* induce male lethality and are responsible in females for incontinentia pigmenti (IP), an X-linked disease mostly characterized by a severe skin inflammation starting at birth. In contrast, hypomorphic mutations of *NEMO*, causing anhidrotic ectodermal dysplasia with immunodeficiency (EDA-ID), affects surviving hemizygous males and is associated with life-threatening impaired immune responses. In both cases, *NEMO* mutations have been identified as affecting either NEMO ubiquitination or NEMO interaction with polyubiquitin chains. For instance, IP-related A323P mutation causes impaired TRAF6-induced polyubiquitination [307] whereas an IP-related internal deletion of NEMO disrupts its interaction with LUBAC subunit SHARPIN [308]. In addition, a large percentage of missense *NEMO* mutations causing EDA-ID affects one of the residues located either in the NUB domain or the zinc finger, producing a NEMO protein with reduced, but not completely abolished, affinity for polyubiquitin [309]. These mutations then provoke suboptimal NF-κB activation for a large set of signaling pathways regulating innate and acquired immunity.

### 9.2. LUBAC Mutations

The importance of M1-linked polyubiquitination in vivo is illustrated in patients bearing mutations in *HOIP* and H*OIL-IL*. In both cases, susceptibility to infection, due to T and B cell defects, and auto-inflammation resulting from complex and cell-specific deregulations of TNF-α and IL-1β/TLR signaling are observed [310,311]. These phenotypes result from impaired formation of M1-linked ubiquitin chains. Although the association of an immunodeficiency with auto-inflammation remains incompletely understood, the immune phenotype of LUBAC mutated patients confirms the essential modulatory function of LUBAC in immune/inflammatory processes.

### 9.3. OTULIN Mutations

Being identified as the only DUB able to remove M1-linked chains from LUBAC substrates, the ubiquitin protease OTULIN when overexpressed should attenuate NF-κB signaling in response to immune-specific receptors, whereas its reduced expression should result in up-regulation of NF-κB targets. This is actually what has been observed on cultured cells [92]. Remarkably, patients carrying *OTULIN* mutations also display such phenotypes. Indeed, the corresponding disease, called either OTULIN-related autoinflammatory syndrome (ORAS) [312] or otulipenia [313], is characterized by the over-production of inflammatory cytokines and autoimmunity associated with excessive M1-linked ubiquitination of LUBAC substrates.

## 10. Conclusions

Over the last fifteen years a wealth of studies has confirmed the critical function of ubiquitin in regulating essential processes such as signal transduction, DNA transcription, endocytosis or cell cycle. Focusing on the ubiquitin-dependent mechanisms of signal regulation and regulation of NF-κB pathways, as done here, illustrates the amazing versatility of ubiquitination in controlling the fate of protein, building of macromolecular protein complexes and fine-tuning regulation of signal transmission. All these molecular events are dependent on the existence of an intricate ubiquitin code that allows the scanning and proper translation of the various status of a given protein. Actually, this covalent addition of a polypeptide to a protein, a reaction that may seem to be a particularly energy consuming process, allows a crucial degree of flexibility and the occurrence of almost unlimited new layers of regulation. This latter point is particularly evident with ubiquitination/deubiquitination events regulating the fate and activity of primary targets often modulated themselves by ubiquitination/deubiquitination events regulating the fate and activity of ubiquitination effectors and so on.

Recurrent features emerge when comparing the various signaling pathways leading to NF-κB described here. In particular, the way in which IKK integrates so many signaling inputs appears to require a rather limited set of proteins (or protein families). In the canonical pathway of NF-κB activation, high variability is seen at distal initiation of signaling but ultimately results in polyubiquitin chain synthesis that attracts, in most situations the TAK1 complex and in all cases the IKK complex. These two elements require distinct kinds of poly-ubiquitin linkages that may be present within the same synthesized chains. The common requirement of TAK1 and IKK in most NF-κB signaling pathways disputes the notion that targeting the TAK1 and/or IKK complexes for therapeutic purpose would be an adequate choice both in term of pathway specificity and adverse side effects. Future research aimed at fully characterizing the specific components/features of each pathway is therefore a prerequisite to efficiently translate this knowledge into valuable clinical options.

Obviously, although our focus in this review has been on ubiquitination processes, low affinity recognition of ubiquitin on modified substrates is not enough to ensure specificity in signal transduction. It is the combined action of UBDs and protein/protein interfaces that ultimately dictates the efficiency of signal transduction. Such specific associations might represent encouraging targets with regard to the aforementioned therapeutic strategies.

Also worth mentioning, are several putative extra layers of complexity that were not discussed above. Their existence is suggested by a disparate collection of data that clearly requires further investigation. First, as already pointed in Section 5.1 concerning the sometime dispensable role of RIPK1, studies suggesting alternative/dual modes of NF-κB activation in major pathways have been published. For instance, it has been claimed that kinase MEKK3 is required for both TNF-α- and IL-1β-dependent NF-κB activation [314,315]. How this enzyme fits into the picture is still unclear but two studies have proposed that it may participate in one of two parallel/sequential pathways of NF-κB activation following IL-1β stimulation [316,317]. All this is reminiscent of what has been observed during TLR4 signaling (see Section 5.2). Further strengthening these models of alternative/redundant modes of activation is the recent publication of Zhang et al. [318] showing TRAF6-dependent NF-κB activation without TAB2/3 subunits. Finally, the most provocative recent discovery, which expands the regulatory function played by polyubiquitination in NF-κB signaling, is the formation of branched chains of ubiquitin (with K48 and K63 links) that may favor TAK1 complex recruitment over CYLD enzymatic activity [319]. This needs to be integrated into signaling processes depending on mixed and unanchored chains of ubiquitin.

To the best of our knowledge the amazingly broad and intricate dependency of NF-κB signaling on ubiquitin has not been observed in any other major signaling pathways. It remains to be seen whether this is a unique property of the NF-κB signaling pathway or only due to a lack of exhaustive characterization of players involved in those other pathways.

Finally, supporting the crucial function of ubiquitin-related processes in NF-κB signaling is their strong evolutionary conservation. Indeed, the immune deficiency (imd) signaling pathway of Drosophila melanogaster, which participates in the fight against pathogens represents the equivalent of a mammalian NF-κB pathway [320]. This pathway shows many similarities to the TNF-R1 signaling pathway, both regarding the nature of the proteins involved and the regulation of their activity through ubiquitin-dependent processes.

## Abbreviations

ABIN	A20-Binding Inhibitor of NF-κB activation
AIP4	Atrophin-1-Interacting Protein 4
ATM	Ataxia Telangiectasia Mutated
BAFF-R	B-cell Activating Factor Receptor
Bcl	B cell lymphoma
BCR	B Cell Receptor
β-TrCP	β-Transducing repeat-Containing Protein
CARD	Caspase Recruitment Domain
CARMA1	CARD-containing a Membrane-Associated Guanylate Kinase (MAGUK) protein 1
CBM	CARMA1/Bcl10/MALT1
CD	Cluster of Differentiation
CDC	Cell Division Cycle
2′-5′-cGAMP	2′-5′-cyclic Guanosine Adenosine MonoPhosphate
cGAS	cyclic Guanosine MonoPhosphate (GMP)-Adenosine MonoPhosphate (AMP) Synthase
CHIP	Carboxy terminus of Hsc70 Interacting Protein
c-IAP	Cellular Inhibitor of Apoptosis Protein
COMMD1	COpper Metabolism MURR1 Domain-containing 1
COP	COnstitutive Photomorphogenesis
CUE	Coupling of Ubiquitin conjugation to Endoplasmic reticulum-associated degradation
CYLD	CYLinDromatosis
DD	Death Domain
DEAD	DEAth effector Domain
DUB	DeUBiquitinase
EDA-ID	anhidrotic ectodermal dysplasia with immunodeficiency
EGLN3	EGL Nine homolog 3
ER	Endoplasmic Reticulum
ERK	Extracellular signal-Regulated Kinase
FADD	Fas-Associated protein with Death Domain
Fbw	F-box/WD repeat-containing protein
FHA	ForkHead Associated
GSK3	Glycogen Synthase Kinase-3
HACE1	HECT domain and Ankyrin repeat Containing E3 ubiquitin protein ligase 1
HECT	Homologous to the E6-AP Carboxyl Terminus
HOIL-1L	Haem-Oxydized IRP2 ubiquitin Ligase 1L
HOIP	HOIL-Interacting Protein
iE-DAP	γ-d-glutamyl-mesoDiAminoPimelic acid
IFN	Interferon
IKK	IκB Kinase
IκB	Inhibitory κB
IL-1βR	Interleukin-1β Receptor
IL1RAP	IL1 Receptor Accessory Protein
Imd	immune deficiency
IP	incontinentia pigmenti
IpaH	Invasion-plasmid antigen-H protein
IRAK	Interleukin-1 Receptor Associated Kinase
iRhom2	inactive Rhomboid 2
JAMM	JAB1/MPN/Mv34 metalloenzyme
KPC1	Kipl ubiquitylation-Promoting Complex 1
LDD	Linear ubiquitin chain Determining Domain
LGP2	Laboratory of Genetics and Physiology 2
LUBAC	Linear UBiquitin chain Assembly Complex
LZ	Leucine Zipper
MAGUK	Membrane-Associated Guanylate Kinase
MALT	Mucosa-Associated Lymphoid Tissue
MALT1	MALT lymphoma associated translocation protein 1
MARCH	Membrane Associated RING-CH
MAVS	Mitochondrial AntiViral-Signaling Protein
MCPIP1	Monocyte Chemotactic Protein-Induced Protein-1
MDA5	Melanoma Differentiation Associated gene 5
Mdm2	Mouse double minute 2 homolog
MDP	Muramyl DiPeptide
MD2	Myeloid Differentiation factor 2
MEKK	Mitogen-activated protein/ERK Kinase Kinase
MEX3C	MEX-3 homolog C (C. elegans)
MJD	Machado-Joseph Diseases protease
MIB2	MIndBomb 2
Mindy	MIU-containing Novel DUB familY
MLKL	Mixed Lineage Kinase domain Like pseudokinase
Myd88	Myeloid differentiation primary response 88
Ndfip1	Nedd4 family interacting protein 1
NEDD8	Neural precursor cell Expressed, Developmentally Down-Regulated 8
NEMO	NF-κB Essential Modulator
NF-κB	Nuclear Factor κB
NIK	NF-κB Inducing Kinase
NLRC	NOD-LRR (Leucine-Rich Repeat) family with CARD
NLRP	Nod-Like Receptor Protein
Nod	Nucleotide-binding oligomerization domain
NSP	Non Structural Protein
NZF	Npl4 Zinc Finger
ORAS	OTULIN-Related Autoinflammatory Syndrome
OUT	Ovarian TUmor proteases
OTULIN	OTU deubiquitinase with LINear linkage specificity
PAMPs	Pathogen Associated Molecular Patterns
PARP-1	Poly[ADP-Ribose (PAR)] Polymerase 1
PPCBP2	Poly(RC) Binding Protein 2
PDLIM2	PDZ and LIM domain 2
PIASy.	Protein Inhibitor of Activated STAT (Signal Transducer and Activator of Transcription) y
PIDD	P53-Induced Protein with a Death Domain
PKC	Protein Kinase C
PPAR	Peroxisome Proliferator-Activated Receptor
PTM	Post-Translational Modifications
PUB	PNGase/UBA or UBX-containing proteins
RBCC	Ring, B-box, Coiled-Coil
Rbx1	RING-box protein 1
Rel	avian Reticuloendotheliosis
RBR	Ring Between Ring fingers
RHIM	RIP Homotypic Interaction Motif
RING	Really Interesting New Gene
RIPK1	Receptor-Interacting serine/threonine-Protein Kinase 1
RLRs	Retinoic-Inducible Gene-I (RIG-I) Like Receptors
RNF	RiNg Finger protein
Sam68	Src-Associated in Mitosis 68 kDa
SCF	Skp, Cullin, F-box
SENP2	SUMO-specific Protease 2
SHARPIN	SHANK-associated RH domain interacting ProteIN
SHIP	SH2-containing Inositol phosphatase
Skp1	S-phase kinase-associated protein 1
Smurf1	Smad ubiquitin regulatory factor 1
SOCS1	Suppressor Of Cytokine Signaling 1
SPATA2	SPermATogenesis-Associated protein 2
Sphk1	Sphingosine kinase 1
STAT	Signal Transducer and Activator of Transcription
STING	STimulator of INterferon Genes
SUMO	Small Ubiquitin-like Modifier
TAB	TAK1 Binding protein
TAD	transcriptional activator domain
TAK1	Tumour growth factor-Activated Kinase 1
TANK	TRAF family member-Associated NF-κB activator
TCR	T cell receptor
TIR	Toll-Interleukin Receptor
TIRAP	Toll-Interleukin Receptor Adaptor Protein
TLR	Toll-Like Receptor
TNFAIP3	TNF Alpha Induced Protein 3
TRADD	Tumor necrosis factor Receptor type 1-Associated Death Domain protein
TRAF	TNF Receptor Associated Factor
TRAM	TRIF-Related Adaptor Molecule
TRIF	TIR domain-containing adaptor-inducing Interferon-β
TRIM	TRIpartite Motif protein
UBA	UBiquitin-Associated
UBASH3A	UBiquitin Associated and SH3 domain containing 3A
Ubc13	Ubiquitin-conjugating 13
UBD	Ubiquitin-Binding Domain
UBE2L3	UBiquitin-conjugating Enzyme E2 L3
UBL	Ubiquitin-Like
UCH	Ubiquitin C-terminal Hydrolases
Uev1A	Ubiquitin-conjugating enzyme variant 1A
ULP	Ubiquitin-Like Proteins
USP	Ubiquitin Specific Protease
WD40	TrpAsp 40 amino acids
WWP	WW domain-containing Protein ligase
XIAP	X-linked Inhibitor of Apoptosis Protein
ZF	Zinc Finger
ZNRF	Zinc aNd Ring Finger
